# Rhabdastrellosides A and B: Two New Isomalabaricane Glycosides from the Marine Sponge *Rhabdastrella globostellata*, and Their Cytotoxic and Cytoprotective Effects

**DOI:** 10.3390/md21110554

**Published:** 2023-10-25

**Authors:** Anastasia B. Kozhushnaya, Sophia A. Kolesnikova, Ekaterina A. Yurchenko, Ekaterina G. Lyakhova, Alexander S. Menshov, Anatoly I. Kalinovsky, Roman S. Popov, Pavel S. Dmitrenok, Natalia V. Ivanchina

**Affiliations:** G.B. Elyakov Pacific Institute of Bioorganic Chemistry, Far Eastern Branch of Russian Academy of Sciences, Pr. 100-Let Vladivostoku 159, 690022 Vladivostok, Russia; kozhushnaia.ab@mail.ru (A.B.K.); eyurch@piboc.dvo.ru (E.A.Y.); elyakhova@inbox.ru (E.G.L.); menshov90@piboc.dvo.ru (A.S.M.); kaaniw@piboc.dvo.ru (A.I.K.); prs_90@mail.ru (R.S.P.); paveldmt@piboc.dvo.ru (P.S.D.); ivanchina@piboc.dvo.ru (N.V.I.)

**Keywords:** isomalabaricanes, glycosides, glycoconjugates, *Rhabdastrella globostellata*, marine sponge, terpenoids, structure elucidation, cytoprotective activity

## Abstract

Investigation of the Vietnamese marine sponge *Rhabdastrella globostellata* led to the isolation of two new polar isomalabaricanes: rhabdastrellosides A (**1**) and B (**2**). Their structures and stereochemistry were elucidated with the application of 1D and 2D NMR, HRESIMS, and HRESIMS/MS methods, as well as chemical modifications and GC–MS analysis. Metabolites **1** and **2** are the first isomalabaricanes with non-oxidized cyclopentane ring in the tricyclic core system. Moreover, having a 3-O-disaccharide moiety in their structures, they increase a very rare group of isomalabaricane glycosides. We report here a weak cytotoxicity of **1** and **2** toward human neuroblastoma SH-SY5Y cells and normal rat H9c2 cardiomyocytes, as well as the cytoprotective activity of rhabdastrelloside B (**2**) at 1 µM evaluated using CoCl_2_-treated SH-SY5Y and H9c2 cells.

## 1. Introduction

Up to now, about 200 isomalabaricanes and their various *nor*-derivatives are known. This class of natural triterpenoids is basically characterized as the C_30_ metabolites constructed of a *trans*-*syn*-*trans*-fused 6,6,5-tricyclic core with a keto function always located at C-12 of ring C, an oxygen-containing group at C-3 of ring A, and a poly-unsaturated or oxidized side chain at C-13. Structural elucidation of isomalabaricanes is often complicated by the ability of some compounds to undergo isomerization of the C-13(14) double bond under light, resulting in a mixture of *Z*/*E*-derivatives [1,2].

Nevertheless, the impressive biological activities demonstrated by isomalabaricane substances [3] make them perspective objects for search and synthesis [4,5]. Among others, their anticancer properties seem to be the most studied so far. In particular, isomeric stellettins A and B at nanomolar concentrations were shown to significantly and selectively act against murine leukemia P388 and human glioblastoma SF295 cells, respectively [6,7]. Lately, the cytoprotective effect of a nanomolar concentration of stellettin B against 6-OHDA-induced cellular damage of SH-SY5Y cells was reported. More interestingly, this triterpenoid was able to reverse a locomotor deficit in an in vivo experiment, using the zebrafish model of Parkinson’s disease [8].

For years, isomalabaricanes were thought to be produced exclusively by marine sponges from the genera *Rhabdastrella*, *Geodia*, *Stelletta*, and *Jaspis*, inhabiting tropical and subtropical sea waters [1,2]. However, the historically used sponge classification, based on the interpretation of morphological characters and frequently limited in phylogenetic information content, led sometimes to the misidentification of sponge samples [9], specifically if they belonged to the abovementioned species-rich genera with similar skeletons [10,11]. Recently, the extensive re-examination of 21 sponge vouchers reported as sources of isomalabaricane triterpenes was provided by Cárdenas et al. As a result, 11 sponge samples assigned previously as *Jaspis* sp., *Jaspis stellifera*, *Stelletta* sp., *Stelletta tenuis*, or *Geodia globostellifera* were found to be misidentified. According to the revision, isomalabaricane triterpenes were isolated only from samples of *R. globostellata*, one currently undescribed species of *Rhabdastrella* sp., and one putative species of *Geodia* [12,13].

We report here a further investigation of the Vietnamese collection of a marine sponge *R. globostellata* previously assigned by us to the *Stelletta* genus [14,15] and re-examined by Dr. Paco Cárdenas. The study on the polar fractions of the EtOH extract of this sponge led to the isolation of two new unusual isomalabaricane glycosides **1** and **2**, whose structures were established by extensive NMR and HR-MS spectral analyses. Cytotoxicities of the isolated compounds against human neuroblastoma SH-SY5Y cells and normal rat cardiomyocytes H9c2, as well as their cytoprotective effects on a CoCl_2_-induced hypoxia-mimic cell model, were evaluated and discussed.

## 2. Results

**Figure 1:**
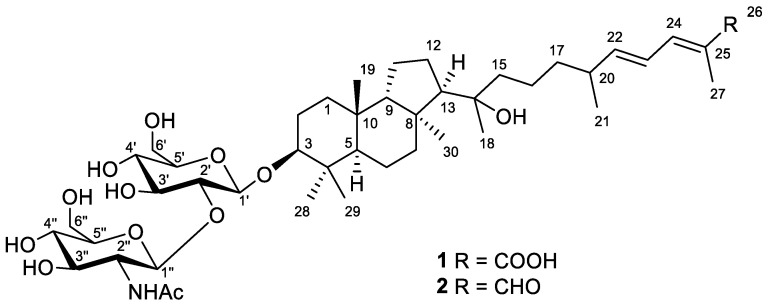
Structures of compounds **1** and **2**.

A frozen specimen of the Vietnamese *R. globostellata* marine sponge, previously reported as *Stelletta* sp. [14,15], was chopped and extracted with EtOH. The extract was concentrated, dissolved in water, and partitioned with EtOAc. Consequent separation of the concentrated EtOAc-soluble materials on Sephadex LH-20 and silica gel led to the nine subfractions of different polarity, as described formerly [15]. New isomalabaricane metabolites **1** and **2** (Figure 1) were isolated from the two most polar subfractions by reversed-phase column chromatography and HPLC (Figure S27).

Compound **1** was obtained as a yellowish powder. Based on the (+)HRESIMS peak at *m*/*z* 884.4730 [M_Na_+Na]^+^, the molecular formula of **1** was deduced as C_44_H_73_NO_14_ (Figure S2). The formation of a cationized molecule with two sodium ions during ESI ionization of compound **1** suggested the presence of a carboxyl group in its structure. Moreover, the peaks found in the (–)HRESIMS/MS spectrum of **1** at *m*/*z* 635.4148 [M–H–C_8_H_13_NO_5_]^−^ and *m*/*z* 473.3621 [M–H–C_8_H_13_NO_5_–C_6_H_10_O_5_]^−^ were attributed to the consequent loss of a one NHAc-hexose and one hexose units from the molecular ion peak at *m*/*z* 838.4944 [M–H]^−^ (Figure S1). Accordingly, the aglycon moiety of **1** corresponded to the formula C_30_H_50_O_4_ with six degrees of unsaturation.

The ^1^H and ^13^C NMR spectra (Table 1 and Table 2, Figures S3 and S4) of **1** confirmed the presence of the detected disaccharide moiety with *β*-configured glycosidic bonds. Thus, characteristic chemical shifts and coupling constants of H-1′ (*δ*_H_ 4.39, d (7.8 Hz)) and H-1″ (*δ*_H_ 4.90, d (8.3 Hz)), carbons C-1′ (*δ*_C_ 105.5), C-1″ (*δ*_C_ 101.9), and NHAc-group (*δ*_H_ 1.99, s; *δ*_C_ 23.0, 174.0) were observed. One carboxyl function (*δ*_C_ 172.3), one disubstituted (*δ*_H_ 5.96, dd (15.0, 8.4 Hz) and 6.38, dd (14.9, 11.5 Hz); *δ*_C_ 125.8, 149.9) and one trisubstituted (*δ*_H_ 7.16, d (11.3 Hz); *δ*_C_ 126.6, 140.2) double bonds satisfied the three degrees of unsaturation calculated for the aglycon part of **1**, while the remaining three enquired its tricyclic framework. Together with this, the ^1^H NMR spectrum showed six additional singlets and one doublet signal of seven methyl groups in aglycon, indicating its triterpenoid nature. Taking into account the previous isolation of a number of isomalabaricane triterpenoids [14,15] from the studied sponge, we suggested the isomalabaricane structure for aglycon of **1** with a classical 6,6,5-tricyclic core and an oxygenated side chain.

**Figure 2:**
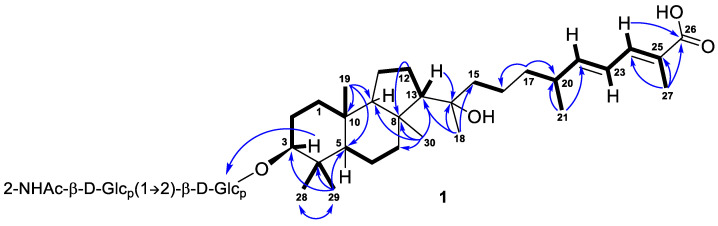
Selected COSY (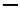) and HMBC (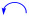) correlations of compound **1**.

Further inspection with the aid of 2D NMR data (Figures S5–S8) allowed us to assign singlet signals to the gem-dimethyl group (*δ*_H_ 1.07, s; *δ*_C_ 29.4 and *δ*_H_ 0.87, s; *δ*_C_ 17.2) at C-4 in ring A, two angular methyls CH_3_-19 (*δ*_H_ 0.97, s; *δ*_C_ 23.8) and CH_3_-30 (*δ*_H_ 1.08, s; *δ*_C_ 32.3), methyl CH_3_-18 (*δ*_H_ 1.12, s; *δ*_C_ 25.6) at oxygen-bearing tertiary carbon C-14 (*δ*_C_ 76.7), and one olefinic methyl CH_3_-27 (*δ*_H_ 1.90, br s; *δ*_C_ 12.7). Doublet resonance at *δ*_H_ 1.05, d (6.8 Hz) was attributed to the CH_3_-21 (*δ*_C_ 20.9) in the side chain of **1**. Indeed, the correlations CH_3_-19/C-5, C-9, C-10; CH_3_-29/CH_3_-28, C-3, C-4, C-5; CH_3_-30/C-7, C-8, C-9, C-13; H*_β_* -12/C-8; H-13/C-14; CH_3_-18/C-13, C-14, C-15; CH_3_-21/C-20, C-22; CH_3_-27/C-24, C-25, C-26; H_2_-17/C-16, C-20; and H-24/C-26 observed in the HMBC spectrum, as well as the revealed ^1^H-^1^H COSY sequences, corresponded to the isomalabaricane skeleton of the aglycon of **1** and established the positions of the disubstituted C-22 double bond, trisubstituted C-24 double bond, and 26-carboxyl group (Figure 2).

Moreover, HMBC from CH_3_-29 to the low-field carbon C-3 (*δ*_C_ 92.5) demonstrated the oxidation in structure **1**, typical of all isomalabaricanes, where the ketone, hydroxyl, or acetate group is usually placed at C-3 [1]. However, the correlation from the proton H-3 at *δ*_H_ 3.21, dd (12.1, 4.8 Hz) to the carbon C-1′ (*δ*_C_ 105.5) of hexose indicated C-3 to be the site of glycosylation in compound **1**. According to the aglycon formula C_30_H_50_O_4_, the other oxygen-containing function, located at C-14, was determined as hydroxyl.

**Figure 3:**
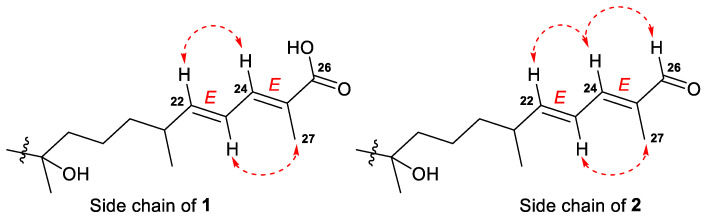
Selected ROESY (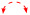) correlations for the side chains of compounds **1** and **2**.

*E*-configurations of the 22- and 24-double bonds in the side chain of **1** were deduced based on two ROESY interactions between H-22/H-24 and H-23/CH_3_-27 (Figure 3 and Figure S13). In a case of a disubstituted 22-double bond, the *trans*-configuration led also from the large coupling constant (*J* = 15.0 Hz) of protons H-22 and H-23.

**Figure 4:**
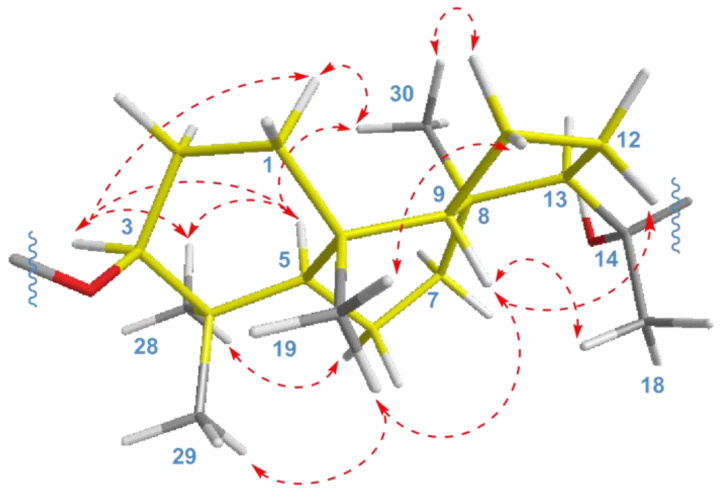
Selected ROESY (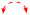) correlations for isomalabaricane core of compounds **1** and **2**.

The key ROESY cross-peaks between CH_3_-28/H-3, H-5; H-5/H-3, CH_3_-30; and CH_3_-19/CH_3_-29, H-9 showed a *trans*-*syn*-*trans* tricyclic system, corresponded to the typical isomalabaricane core and *β*-orientation of the glycoside moiety at C-3 (Figure 4). Additional correlations H_α_-1/H-3, CH_3_-30; H*_β_*-2/CH_3_-19; H_α_-6/CH_3_-28; H*_β_*-11/CH_3_-19; H_α_-11/CH_3_-30; and H*_β_*-12/H-9 gave the spatial orientations of the corresponding protons. Whereas the ROESY coupling between H-9 and methyl CH_3_-18 evidenced a *β*-oriented side chain in the aglycon part of **1**, and hence an α-oriented proton, H-13. Due to the degradation of the aglycon during hydrolysis, we were unable to obtain its chemical derivatives and establish the C-14 and C-20 absolute configurations.

As a result, the 22*E*,24*E*-3*S*,5*R*,8*S*,9*S*,10*R*,13*R*,14*ξ*,20*ξ* stereochemistry was deduced for the aglycon of compound **1**. We believe that it can be taken as absolute, since a number of isomalabaricanes previously isolated from the studied sponge have the same absolute configurations of asymmetric centers, belonging to the tricyclic system [14,15].

**Figure 5:**
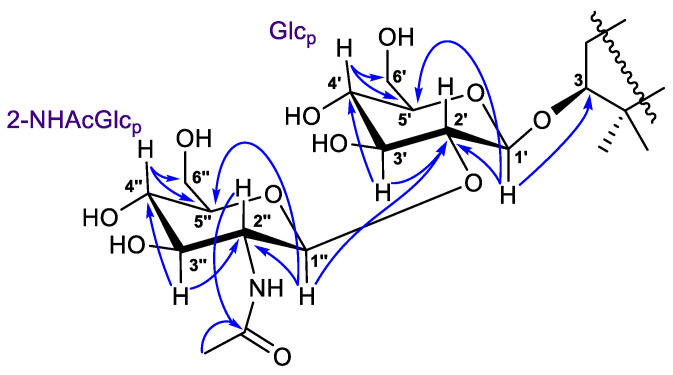
Selected HMBC (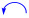) correlations for carbohydrate chain in compounds **1** and **2**.

The ^1^H-^1^H COSY, HSQC, HMBC, and TOCSY experiments led to the assignment of all the proton and carbon signals of the 3-*O*-carbohydrate chain in **1** (Table 2, Figures S5–S10). In detail, the ^1^H-^1^H COSY correlations revealed two isolated spin systems, each consisting of five methines and one methylene. The HMBCs from deshielded protons (Figure 5) showed the C-1′–C-6′ and C-1″–C-6″ carbon sequences in monosaccharides and the position of the NHAc-group at C-2″ (*δ*_C_ 58.2). The (1→2)-connection of sugar units in **1** was indicated by the HMBC cross-peak from the proton H-1″ to the C-2′ carbon.

Coupled system of the resonating protons H-1′/H-2′/H-3′/H-4′/H-5′ in the 2D TOCSY spectrum of **1** (Figures S9 and S10) corresponded to their relative *trans*-positions, suggesting the pyranose form of glucose at C-3. Moreover, the chemical shifts and coupling constants of protons H-1′–H-6′, as well as the carbon signals, most closely resembled those of a 3-*O*-glucose of the reported spirostanol glycoside from the seeds of *Hyoscyamus niger* L. [16]. Based on the NMR observed above and mass-spectrometry evidence, similarity of the corresponding NMR signals with the literature data [17], and H-1″/H-2″/H-3″/H-4″/H-5″ 2D TOCSY interactions, the terminal sugar unit was identified as 2-N-acetyl-glucosamine. As a result, the 3-*O*-[2-NHAc-*β*-Glc_p_(1→2)-*β*-Glc_p_] carbohydrate chain was determined for the structure of a new isomalabaricane glycoside **1**, named rhabdastrelloside A (**1**).

**Figure 6:**
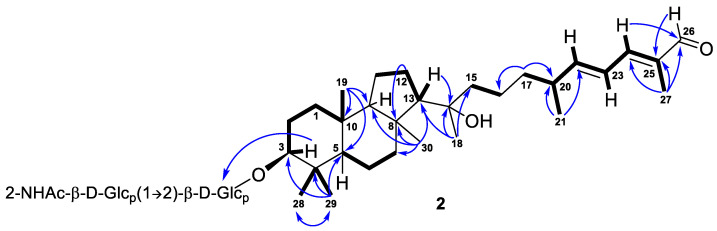
Selected COSY (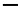) and HMBC (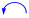) correlations of compound **2**.

Compound **2**, isolated as a yellowish powder, has a molecular formula C_44_H_73_NO_13_ as it was established on the basis of (+)HRESIMS peak at *m*/*z* 846.4973 [M+Na]^+^ (Figure S14). Two peaks found in the (–)HRESIMS/MS spectrum of **2** at *m*/*z* 619.4226 [M–H–C_8_H_13_NO_5_]^−^ and *m*/*z* 457.3695 [M–H–C_8_H_13_NO_5_–C_6_H_10_O_5_]^−^ (Figure S13), as well as the identical ^1^H and ^13^C NMR signals (Table 2, Figures S15 and S16), indicated the same 3-*O*-[2-NHAc-*β*-Glc_p_(1→2)-*β*-Glc_p_] carbohydrate chain in structure **2**. The aglycon moiety of glycoside **2** corresponded to the formula C_30_H_50_O_3_ and contained one fewer oxygen atom than aglycon of **1**. Moreover, it also showed very similar chemical shifts and coupling constants except those attributed to the side chain atoms (Table 1). Thus, the low-field singlet at *δ*_H_ 9.38 and the carbon shift at *δ*_C_ 197.0 in the spectra of **2**, along with mass spectrometry data, evidenced the presence of a 26-aldehyde function in the side chain of **2**, instead of the 26-carboxyl group in **1**. Key ^1^H-^1^H COSY, HSQC, HMBC, and ROESY (Figures S17–S20) correlations confirmed the proposed structure of a new isomalabaricane glycoside **2**, named rhabdastrelloside B (**2**). In particular, the HMBC cross-peaks H-24/C-26 and H-26/C-25 completely satisfied the C-26 position of the aldehyde (Figure 6) as well as the ROESY interactions between protons H-22/H-24, H-24/H-26, and H-23/CH_3_-27 detected *E*-configurations of 22,24-double bonds in **2** (Figure 3).

We used a portion of compound **2** to establish the stereochemistry of the disaccharide chain in both new isomalabaricane glycosides. A hydrolysis of **2**, in the presence of trifluoroacetic acid, gave a sum of sugars treated further with *R*-(–)-2-octanol. The analysis of acetylated 2-octylglycosides was carried out by GC–MS using the corresponding derivatives prepared from standard monosaccharides following the same procedure (Figures S23–S26). As a result, D-configurations were determined for glucose and N-acetyl-glucosamine units in a 3-*O*-[2-NHAc-*β*-D-Glc_p_(1→2)-*β*-D-Glc_p_] carbohydrate chain.

The cytotoxicity of compounds **1** and **2** against human neuroblastoma SH-SY5Y cells and normal rat cardiomyocytes H9c2, as well as their effect on the viability of CoCl_2_-treated cells, was investigated. Cobalt chloride (II) induces hypoxia-like cell damage and is widely used to search for compounds with cytoprotective properties in hypoxia-mimic conditions [18]. At a concentration of 100 µM, both compounds decreased the cells’ viability by 22.9% and 13.5% for SH-SY5Y, and 35.2% and 21.8% for H9c2 cells, respectively. Moreover, both compounds were toxic for H9c2 cells at 10 µM and decreased their viability by 21.7% and 20.4%, respectively. Accordingly, to investigate the cytoprotective properties of the compounds, they were used at a concentration of 1 µM.

**Figure 7:**
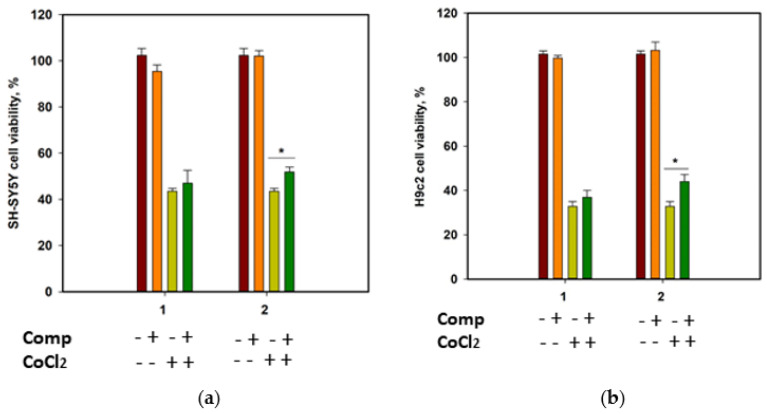
Influence of compounds **1** and **2** on the viability of SH-SY5Y (**a**) and H9c2 (**b**) cells. Both compounds were used at a concentration of 1 µM. Cobalt chloride (II) was used at 500 µM. All experiments were carried out in triplicates. The data are presented as a mean ± standard error of mean. Asterisk * indicates the significance of differences between variants with *p*-value ≤ 0.05.

Since SH-SY5Y neuroblastoma cells are more sensitive to CoCl_2_-induced damage than H9c2 cardiomyocytes [19], the viability of CoCl_2_-treated SH-SY5Y cells was measured after 24 h, and the viability of CoCl_2_-treated H9c2 cells was estimated after 48 h (Figure 7). Cobalt chloride (II) decreased the viability of SH-SY5Y and H9c2 cells by 56.5% and 67.3%, respectively. Rhabdastrelloside A (**1**) did not show notable effects, while rhabdastrelloside B (**2**) statistically increased the viability of CoCl_2_-treated SH-SY5Y and H9c2 cells by 19.3% and 34.1%, respectively.

## 3. Discussion

New compounds **1** and **2** expand a relatively small group of glycosides from marine sponges, which previously included about 144 metabolites (94 tetracyclic triterpenoid glycosides and 50 steroid ones), isolated by the end of 2022. Natural products of this chemical class were found in the sponges belonging to five orders, where they were present in many families and species without apparent dependence on environmental or geographical factors. The most frequently, triterpene and steroid glycosides, were isolated from sponges of the Tetractinellida (suborder Astrophorina) and Poecilosclerida orders. [20] The first isomalabaricane triterpene glycoside, stelliferin riboside [21], was reported in 2001 as a metabolite isolated from *Geodia globostellifera*, recently re-identified as *R. globostellata* (Tetractinellida order) [12]. Later, stelliferin riboside [22,23], as well as its 3-*O*-deacetyl analogue and 13*E*-isomer [22], were found in Indonesian and Fijian collections of *R. globostellata*. These three compounds represent the only previously known glycosylated isomalabaricanes. Being 22-*O*-ribopyranosides with a glycosylation site in the side chain of the triterpene aglycon, they differ significantly in their structure from the new compounds **1** and **2** isolated by us.

Even more intriguing is the absence of the 12-keto function, common to all known isomalabaricanes, in the structures of rhabdastrellosides A (**1**) and B (**2**). Here, we can draw some parallels in the biosynthesis of spongial isomalabaricanes and malabaricane triterpenoids found in several terrestrial plants, fungi, ferns, and sediments. Both triterpenoid skeletons look similar, but sponge metabolites differ from malabaricanes in the *trans*–*syn*–*trans* conjunction of the rings in a core system, while malabaricanes possess *trans*–*anti*–*trans* stereochemistry [1]. Since the discovery of malabaricol in 1967 [24], only 48 triterpenoids of the malabaricane class were reported [1,25]. However, this small group of natural compounds includes 3-*O*-glycosides, three compounds found in an endemic species *Adesmia aconcaguensis* from the central Chilean Andes [26], and twenty astramalabaricosides A–T isolated from the roots of *Astragalus membranaceus* var. *mongholicus* in 2022 [25]. All reported malabaricane glycosides have carbohydrate chains comprising one to five monosaccharide units. Moreover, like isomalabaricanes, they are oxidized at C-12 with the presence of a hydroxy function in a core instead of a 12-ketone.

**Figure 8:**
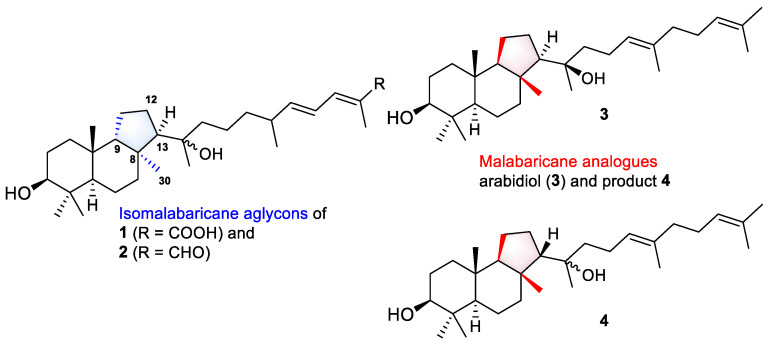
Aglycons of new isomalabaricane glycosides **1** and **2**, and malabaricane analogues **3** and **4** biosynthesized by modified arabidiol synthase AmAS from *A*. *membranaceus* [27].

In addition, a very recent study of Song et al. on an *A. membranaceus* describes the characterization of a new arabidiol synthase AmAS, which is responsible for the formation of the malabaricane-type backbone in the biosynthesis of astramalabaricosides. Site-directed mutagenesis of the key residue IIH725-727 with VFN led to seven triterpenoidal products, including arabidiol (**3**) and compound **4**, which are partially malabaricane analogues of the aglycon parts in **1** and **2** (Figure 8). Further experimental glycosylation of triterpenoidal substrate **3** with plant glycosyltransferases from *A*. *membranaceus* led to the products containing 3-*O*-mono- and disaccharide chains [27].

Based on the structural similarity between the investigated malabaricanes and new isomalabaricanes **1** and **2**, we may suggest that glycosylation is a prior or parallel step to oxidation at C-12 in the biosynthesis of both types of triterpenoids. We also expect the presence of the analogues enzymatic complex of the oxidosqualene cyclase able to form the isomalabaricane skeleton and corresponding glycosyltransferases in *R. globostellata* marine sponges.

As for the biological activity of known isomalabaricane glycosides, the moderate cytotoxicity of stelliferin riboside against A2780 ovarian cancer cells (IC_50_ = 38.1 μg/mL) [21], and the ability of this compound to stabilize the binding of DNA polymerase β to DNA, inducing 29% binding at 28 μg/mL [23], were reported. Moreover, stelliferin riboside and 3-*O*-deacetyl-13*Z*-stelliferin riboside were shown to possess extremely high and selective activity against the mouse lymphoma L5178Y cancer cell line with ED_50_ values of 0.22 and 2.4 nM, respectively [22].

Our results detected a weak cytotoxicity of the new isomalabaricane glycosides **1** and **2** against SH-SY5Y and H9c2 cells, and the ability of rhabdastrelloside B (**2**) at nontoxic concentration to increase the viability of CoCl_2_-treated SH-SY5Y and H9c2 cells. Obviously, the presence of the 26-aldehyde function in the side chain enhances the cytoprotective activity of rhabdastrelloside B (**2**) compared to rhabdastrelloside A (**1**).

Similar to hypoxia induced by O_2_ deprivation, toxic effects of CoCl_2_ treatment include oxidative stress with inhibition of DNA repair, typical apoptotic changes, and mitochondrial DNA damage [28]. This is mediated by the stabilization of the hypoxia-induced transcriptional factor HIF1α, which, in turn, triggers a cascade of molecular reactions including the activation of the Nrf2-dependent antioxidant defense system [29]. The reported effect of stellettin B, which induced nuclear translocation of Nrf2 resulting in overexpression of antioxidant enzymes in 6-OHDA-treated SH-SY5Y cells [8], allows us to suggest that the new isomalabaricane glycoside **2** may also act as an antioxidant. However, this assumption requires detailed study.

## 4. Materials and Methods

### 4.1. General Experimental Procedures

Optical rotations were measured on Perkin-Elmer 343 digital polarimeter (Perkin Elmer, Waltham, MA, USA). UV-spectra were registered on a Shimadzu UV-1601PC spectrophotometer (Shimadzu Corporation, Kyoto, Japan). ECD spectra were recorded on a Chirascan plus instrument (Applied Photophysics Ltd., Leatherhead, UK). ^1^H NMR (500.13 MHz, 700.13 MHz) and ^13^C NMR (125.75 MHz, 176.04 MHz) spectra were recorded in CD_3_OD on Bruker Avance III HD 500 and Bruker Avance III 700 spectrometers (Bruker BioSpin, Bremen, Germany). The ^1^H and ^13^C NMR chemical shifts were referenced to the solvent peaks at δ_H_ 3.30 and δ_C_ 49.0 for CD_3_OD. HRESIMS analyses were performed using a Bruker Impact II Q-TOF mass spectrometer (Bruker, Bremen, Germany). The operating parameters for ESI were as follows: a capillary voltage of 3.5 kV, nebulization with nitrogen at 0.8 bar, dry gas flow of 7 L/min at a temperature of 200 °C. The mass spectra were recorded within *m*/*z* mass range of 100–1500. The instrument was operated using the otofControl (ver. 4.1, Bruker Daltonics) and data were analyzed using the DataAnalysis Software (ver. 4.4, Bruker Daltonics). GC–MS analyses were carried out on a Hewlett Packard HP6890 GC System (Hewlett-Packard Company, Palo Alto, CA, USA), with an HP-5MS (J&W Scientific, Folsom, CA, USA) capillary column (30 m × 0.25 mm, 0.25 μm). The carrier gas was helium (flow rate 1.0 mL/min), the injector temperature was 270 °C, the ionizing voltage was 70 eV, and the temperature program was 100 °C (0.5 min) −5 °C/min −250 °C (10 min). Column chromatography was performed on Sephadex LH-20 (25–100 µm, Pharmacia Fine Chemicals AB, Uppsala, Sweden), silica gel (KSK, 50–160 mesh, Sorbfil, Krasnodar, Russia), and YMC ODS-A (12 nm, S-75 um, YMC Co., Ishikawa, Japan). HPLC was carried out using an Agilent 1260 Infinity II chromatograph equipped with a differential refractometer (Agilent Technologies, Santa Clara, CA, USA). The reversed-phase columns YMC-Pack ODS-A (YMC Co., Ishikawa, Japan, 10 × 250 mm, 5 µm and 4.6 mm × 250 mm, 5 µm) and Discovery HS F5-5 (SUPELCO Analytical, Bellfonte, PA, USA, 10 × 250 mm, 5 µm) were used for HPLC. Yields are based on dry weight (212.1 g) of the sponge sample.

### 4.2. Animal Material

The sponge sample (1.3 kg) was collected by SCUBA diving at a depth of 7–12 m near Cham Island (15°54.3′ N, 108°31.9′ E) in the Vietnamese waters of the South China Sea during the 38th cruise of R/V “Academik Oparin” in May 2010. The species was previously identified and described as *Stelletta* sp. [14]. According to an extensive revision of sponge specimens provided by Dr. Paco Cárdenas (Department of Pharmaceutical Biosciences, Uppsala University, Sweden), this sponge was re-identified as *Rhabdastrella globostellata* [12]. A voucher specimen (PIBOC O38-301) has been deposited at the collection of marine invertebrates of the G.B. Elyakov Pacific Institute of Bioorganic Chemistry FEB RAS, Vladivostok, Russia.

### 4.3. Extraction and Isolation

The frozen sponge EtOH extract (1.7 L × 3) was concentrated (52.5 g), dissolved in distilled H_2_O (100 mL), and partitioned with EtOAc (100 mL × 3) (Figure S27). The resulting EtOAc extracts gave a dark brown gum (15.7 g). Separation of this sum on a Sephadex LH-20 column (2 × 95 cm, CHCl_3_/EtOH, 1:1) yielded three fractions. The major fraction 2 (10.2 g) was divided into nine subfractions using step-wise gradient silica gel column chromatography (4 × 15 cm, CHCl_3_→EtOH) as described previously [15]. The two most polar subfractions F9 (182.5 mg) and F8 (324.7 mg), eluted with 100% EtOH, were separated using step-wise gradient reversed-phase column chromatography (1 × 5 cm, YMC-Pack ODS-A, 10% EtOH→100% EtOH) and HPLC. Pure compound **2** (1.0 mg, 0.001%) along with subfraction containing compound **1** were isolated from the subfraction F9.3 (76.3 mg, eluted with 60% EtOH) by HPLC (YMC-Pack ODS-A) in 80% MeOH. Compound **1** (1.4 mg, 0.001%) was further purified by HPLC (Discovery HS F5-5) in 80% MeOH. Separation of fraction F8.3 (284.7 mg, eluted with 60% EtOH) by HPLC (YMC-Pack ODS-A) in 80% MeOH and further re-chromatography (Discovery HS F5-5) in 80% MeOH gave new portions of compounds **1** (1.1 mg) and **2** (1.8 mg).

### 4.4. Compound Characteristics

Rhabdastrelloside A (**1**): Yellowish powder; [*α*]_D_^25^ 0.0 (*c* 0.1, MeOH); UV (EtOH) λ_max_ (log ε) 260 (4.14) nm; ECD (*c* 8.3 × 10^−4^ M, EtOH) *λ*_max_ (Δ*ε*) 205 (–0.87), 257 (1.10), 326 (–0.48), 335 (–0.48), 343 (–0.47), 460 (–0,08) nm; ^1^H and ^13^C NMR data (CD_3_OD), Table 1 and Table 2; (+)HRESIMS *m*/*z* 884.4730 [M_Na_+Na]^+^ (calcd for C_44_H_73_NO_14_Na_2_ 884.4716); (–)HRESIMS *m*/*z* 838.4944 [M–H]^−^ (calcd for C_44_H_72_NO_14_ 838.4931); (–)HRESIMS/MS of the ion [M–H]^−^ at *m*/*z* 838.4929: 635.4148 [M–H–C_8_H_13_NO_5_]^−^, 473.3621 [M–H–C_8_H_13_NO_5_–C_6_H_10_O_5_]^−^.

Rhabdastrelloside B (**2**): Yellowish powder; [*α*]_D_^20^–27.0 (*c* 0.1, MeOH); UV (EtOH) λ_max_ (log ε) 281 (4.11) nm; ECD (*c* 1.2 × 10^−3^ M, EtOH) *λ*_max_ (Δ*ε*) 207 (–0.20), 227 (–0.17), 271 (0.41), 278 (0.41), 287 (0.39), 326 (–0.02) nm; ^1^H and ^13^C NMR data (CD_3_OD), Table 1 and Table 2; (–)HRESIMS *m*/*z* 822.5033 [M–H]^−^ (calcd for C_44_H_72_NO_13_ 822.5009); (+)HRESIMS *m*/*z* 846.4973 [M+Na]^+^ (calcd for C_44_H_73_NO_13_Na 846.4974); (–)HRESIMS/MS of the ion [M–H]^−^ at *m*/*z* 822.5028: 619.4226 [M–H–C_8_H_13_NO_5_]^−^, 457.3695 [M–H–C_8_H_13_NO_5_–C_6_H_10_O_5_]^−^.

### 4.5. Acid Hydrolysis and Determination of Absolute Configurations of Monosaccharides

Acid hydrolysis of compound **2** (1.4 mg) was carried out in a solution of 2 M trifluoroacetic acid (TFA, 750 μL) in a sealed vial using a water bath at 100 ^°^C for 2 h. The H_2_O layer was washed with *n*-hexane (3 × 750 μL) and concentrated under reduced pressure. One drop of concentrated TFA and 500 μL of *R*-(–)-2-octanol (Sigma Aldrich) were added to the obtained mixture in the sealed vial and then heated on a glycerol bath at 130 °C for 6 h. The solution was evaporated using a Smart Evaporator (BioChromato, Japan) and the resulting products were treated with a mixture of pyridine/acetic anhydride (1:1, 400 μL) for 24 h at room temperature. The acetylated 2-octylglycosides were purified by silica gel (2 × 0.5 cm) flash chromatography in 100% MeOH and then analyzed by GC–MS using the corresponding authentic samples prepared by the same procedure (Figures S23–S27) [30]. The retention times (t_R_) and EIMS spectra showed for the components of the hydrolysate corresponded to those of the authentic samples and were as follows:

D-glucose: t_R_ 28.05 min, *m*/*z* (%): 354 (2), 331 (97), 315 (13), 289 (4), 259 (4), 242 (17), 229 (11), 215 (9), 203 (27), 169 (98), 157 (45), 143 (100), 127 (42), 112 (67), 98 (99), 81 (42), 71 (46), 57 (54); t_R_ 28.51 min, *m*/*z* (%):354 (2), 331 (13), 289 (5), 271 (2), 242 (43), 229 (9), 215 (7), 200 (55), 182 (9), 169 (58), 157 (100), 140 (57), 127 (18), 115 (96), 98 (95), 81 (32), 71 (25), 57 (34); t_R_ 28.80 min, *m*/*z* (%): 354 (2), 331 (36), 289 (3), 271 (5), 242 (42), 229 (8), 200 (45), 182 (9), 169 (100), 157 (89), 140 (46), 127 (21), 115 (87), 98 (74), 81 (24), 71 (25), 57 (33) and t_R_ 29.13 min, *m*/*z* (%):354 (2), 331 (100), 315 (9), 289 (2), 259 (2), 242 (11), 227 (9), 215 (5), 203 (16), 183 (12), 169 (71), 157 (27), 143 (60), 127 (23), 115 (46), 98 (62), 81 (25), 71 (36), 57 (40);

N-acetyl-D-glucosamine: t_R_ 31.51 min, *m*/*z* (%): 355 (4), 330 (8), 318 (3), 288 (4), 259 (5), 241 (52), 226 (10), 210 (10), 199 (38), 181 (40), 168 (20), 156 (66), 139 (72), 126 (22), 114 (100), 96 (49), 84 (27), 72 (20), 57 (18).

### 4.6. Cell Culture

The human neuroblastoma SH-SY5Y cells were purchased from ATCC (Manassas, VA, USA). The rat cardiomyocytes H9c2 cells were kindly provided by Prof. Dr. Gunhild von Amsberg from Martini-Klinik Prostate Cancer Center, University Hospital Hamburg-Eppendorf, Hamburg, Germany. The cells were cultured in DMEM media (BioloT, St. Petersburg, Russia) with 10% fetal bovine serum (BioloT, St. Petersburg, Russia) and 1% penicillin/streptomycin (BioloT, St. Petersburg, Russia) at 37 °C and 5% CO_2_.

### 4.7. Cell Viability Assay

The SH-SY5Y and H9c2 cells were seeded at concentrations of 5 × 10^3^ cell/well and 3 × 10^3^ cell/well, respectively, and the experiments were started after 24 h. The compounds at concentrations up to 100 µM were added into the wells for 48 h, and the viability of the cells was measured by an MTT (3-(4,5-dimethylthiazol-2-yl)-2,5-diphenyltetrazolium bromide) assay, which was performed according to the manufacturer’s instructions (Sigma-Aldrich, Munich, Germany). All compounds were dissolved with DMSO so that the final concentration of DMSO in the cell culture was not more than 1%. Moreover, DMSO was used as a control. The results were presented as a percentage of the control data.

### 4.8. Cardioprotective Activity of Compounds in CoCl_2_-Mimic Hypoxia

The cells were treated with a dH_2_O-solution of CoCl_2_ at a concentration of 500 µM for 1 h. Then, compounds at a concentration of 1 µM were added for 23 h (SH-SY5Y) or 47 h (H9c2). The viability of the treated cells was measured by an MTT assay.

### 4.9. Statistical Data Evaluation

All the data were obtained in three independent replicates, and the calculated values were expressed as a mean ± standard error mean (SEM). A Student’s *t*-test was performed using SigmaPlot 14.0 (Systat Software Inc., San Jose, CA, USA) to determine the statistical significance. The differences were considered statistically significant at *p* < 0.05.

## 5. Conclusions

To conclude, the present article describes the structure elucidation of two new polar isomalabaricane glycosides, rhabdastrellosides A (**1**) and B (**2**), found in the Vietnamese marine sponge *R. globostellata*. These two compounds represent the first 12-dihydroisomalabaricanes. Moreover, having the 3-*O*-disaccharide carbohydrate chain in their structures, they complement the very rare group of isomalabaricane glycosides in addition to three previously known monoribosides. We report here a weak cytotoxicity of metabolites **1** and **2** at 10 µM toward normal rat cardiomyocytes H9c2, as well as the cytoprotective effect of rhabdastrelloside B (**2**) at 1 µM measured using CoCl_2_-treated neuroblastoma cells SH-SY5Y and cardiomyocytes H9c2.

## Figures and Tables

**Table 1:** ^1^H and ^13^C NMR data of aglycon parts of compounds **1** and **2** in CD_3_OD.

No. ^1^	1 (700 and 176 MHz)		2 (500 and 126 MHz)	
	*δ*_H_ mult (*J* in Hz)	*δ*_C_	*δ*_H_ mult (*J* in Hz)	*δ*_C_
1*α* 1*β*	1.47, m 1.37, m	35.3	1.47, m 1.37, m	35.3
2*α* 2*β*	2.03, m 1.77, m	29.0	2.03, m 1.77, m	29.0
3*α*	3.21, dd (12.1, 4.8)	92.5	3.21, dd (11.7, 4.9)	92.4
4		40.9		40.9
5*α*	1.61, m	48.0	1.62, m	48.0
6*α* 6*β*	1.58, m 1.37, m	19.4	1.58, m 1.37, m	19.4
7a 7b	2.61, m 1.35, m	34.1	2.63, m 1.36, m	34.1
8		44.3		44.3
9*β*	1.46, m	57.3	1.46, m	57.3
10		36.7		36.7
11*α* 11*β*	1.33, m 1.50, m	22.6	1.33, m 1.50, m	22.6
12*α* 12*β*	1.48, m 1.75, m	26.2	1.48, m 1.75, m	26.3
13*α*	1.58, m	60.8 ^2^	1.57, m	60.8 ^2^
14		76.7		76.7
15	1.48, m (2H)	42.2 ^2^	1.47, m (2H)	42.2 ^2^
16	1.36, m 1.48, m	22.7	1.35, m 1.48, m	22.7
17	1.35, m (2H)	38.6	1.38 m (2H)	38.5
18	1.12, s	25.6 ^2^	1.12, s	25.6 ^2^
19	0.97, s	23.8	0.97, s	23.7
20	2.32, m	38.7	2.38, m	39.0
21	1.05, d (6.8)	20.9	1.08, d (7.0)	20.6
22	5.96, dd (15.0, 8.4)	149.9	6.22, dd (15.1, 8.3)	153.3
23	6.38, dd (14.9, 11.5)	125.8	6.59, dd (15.1, 11.1)	125.6
24	7.16, d (11.3)	140.2	6.98, d (11.1)	151.5
25		126.6 ^2^		137.2
26		172.3	9.38, s	197.0
27	1.90, s	12.7	1.80, s	9.3
28	1.07, s	29.4	1.07, s	29.4
29	0.87, s	17.2	0.87, s	17.2
30	1.08, s	32.3	1.08, s	32.3

^1^ Assignments were made with the aid of HSQC, HMBC, and ROESY data; ^2^ The values were found from HMBC experiment.

**Table 2:** ^1^H and ^13^C NMR data of disaccharide moieties of **1** and **2** in CD_3_OD.

No. ^1^	1 (700 and 176 MHz)		2 (500 and 126 MHz)	
	*δ*_H_ mult (*J* in Hz)	*δ*_C_	*δ*_H_ mult (*J* ^2^ in Hz)	*δ*_C_
1′	4.39, d (7.8)	105.5	4.39, d (7.8)	105.4
2′	3.59, t (8.5)	79.3	3.58, t (8.6)	79.3
3′	3.43, t (8.8)	79.3	3.43, t (8.8)	79.3
4′	3.25, m	72.0	3.24, m	72.0
5′	3.24, m	77.7	3.24, m	77.7
6′	3.84, m 3.63, m	62.9	3.84, d (12.8) 3.63, dd (11.9, 5.7)	62.9
1″	4.90, d (8.3)	101.9	4.90, d (8.3)	102.0
2″	3.65, dd (10.3, 8.3)	58.2	3.63, dd (10.1, 8.6)	58.2
3″	3.40, dd (10.3, 8.7)	76.7	3.41, t (9.4)	76.7
4″	3.14, t (9.3)	72.9	3.15, t (9.3)	72.9
5″	3.25, m	78.3	3.25, ddd (9.4, 7.5, 2.5)	78.3
6″	3.85, m 3.57, dd (12.0, 7.6)	63.6	3.85, dd (12.0, 2.5) 3.57, dd (11.8, 7.4)	63.6
NHAc	1.99, s	23.0 174.0	1.98, s	23.0 174.0

^1^ Assignments were made with the aid of ^1^H-^1^H COSY, HSQC, HMBC, and TOCSY data; ^2^ Coupling constants were taken from 1D TOCSY spectra (Figure S21).

## Data Availability

Not applicable.

## Supplementary Materials

The following are available online at https://www.mdpi.com/article/10.3390/md21110554/s1, Figures S1 and S2: HRESIMS and MS/MS spectra for rhabdastrelloside A (**1**), Figures S3–S11: ^1^H and ^13^C NMR, HSQC, HMBC, COSY, 2D TOCSY, and ROESY spectra of rhabdastrelloside A (**1**) in CD_3_OD (700 MHz), Figure S12: UV and ECD spectra (EtOH) of rhabdastrelloside A (**1**), Figures S13 and S14: HRESIMS and MS/MS spectra for rhabdastrelloside B (**2**), Figures S15–S21: ^1^H and ^13^C NMR (500 MHz), HSQC (500 MHz), HMBC (700 MHz), COSY (500 MHz), 1D TOCSY (500 MHz), and ROESY (500 MHz) spectra of rhabdastrelloside B (**2**) in CD_3_OD, Figure S22: UV and ECD spectra (EtOH) of rhabdastrelloside B (**2**), Figures S23–S27: determination of absolute configurations of monosaccharides: GC–MS profiles and EIMS spectra of detected and standard monosaccharides, Figure S28: the isolation scheme.
