# Supplementary material for: Rhabdastrellosides A and B: Two New Isomalabaricane Glycosides from the Marine Sponge Rhabdastrella globostellata, and Their Cytotoxic and Cytoprotective Effects
Source: Mar Drugs. 2023 Oct 25;21(11):554. doi: 10.3390/md21110554 (PMC10672615; doi:10.3390/md21110554)
Supplement: Supplementary file 1 [file marinedrugs-21-00554-s001.zip › marinedrugs-2660646.pdf]

Supplementary Materials

Rhabdastrellosides A and B, isomalabaricane glycosides from the marine sponge *Rhabdastrella globostellata*, and their cytotoxic and cytoprotective effects

Anastasia B. Kozhushnaya [1,⊥], Sophia A. Kolesnikova [1,⊥,*], Ekaterina A. Yurchenko [1], Ekaterina G. Lyakhova [1], Alexander S. Menshov [1], Anatoly I. Kalinovsky [1], Roman S. Popov [1], Pavel S. Dmitrenok [1] and Natalia V. Ivanchina [1]

[1] G.B. Elyakov Pacific Institute of Bioorganic Chemistry, Far Eastern Branch of Russian Academy of Sciences, Pr. 100-let Vladivostoku 159, 690022 Vladivostok, Russia; kozhushnaia.ab@mail.ru (A.B.K.); eyurch@piboc.dvo.ru (E.A.Y.); elyakhova@inbox.ru (E.G.L.); menshov90@piboc.dvo.ru (A.S.M.); kaaniw@piboc.dvo.ru (A.I.K.); prs_90@mail.ru (R.S.P.); paveldmt@piboc.dvo.ru (P.S.D.); ivanchina@piboc.dvo.ru (N.V.I.)

⊥ A.B.K. and S.A.K. contributed equally.

* Correspondence: sovin81@inbox.ru (S.A.K.); Tel.: +7-423-231-1168

Contents:

S1 (−)HRESIMS of Rhabdastrelloside A (**1**) and MS/MS spectrum of its $[M - H]^-$ precursor ion at *m/z* 838
S2 HRESIMS (Positive Ion Mode) of Rhabdastrelloside A (**1**)
S3 ^{1}H NMR Spectrum of Rhabdastrelloside A (**1**) in CD_3OD (700 MHz)
S4 ^{13}C NMR Spectrum of Rhabdastrelloside A (**1**) in CD_3OD (700 MHz)
S5 HSQC Spectrum of Rhabdastrelloside A (**1**) in CD_3OD (700 MHz)
S6 HMBC Spectrum of Rhabdastrelloside A (**1**) in CD_3OD (700 MHz)
S7 COSY Spectrum of Rhabdastrelloside A (**1**) in CD_3OD (700 MHz)
S8 Expanded COSY Spectrum of Rhabdastrelloside A (**1**) in CD_3OD (700 MHz)
S9 2D TOCSY Spectrum of Rhabdastrelloside A (**1**) in CD_3OD (700 MHz)
S10 Expanded 2D TOCSY Spectrum for disaccharide moiety in Rhabdastrelloside A (**1**) in CD_3OD (700 MHz)
S11 ROESY Spectrum of Rhabdastrelloside A (**1**) in CD_3OD (700 MHz)
S12 UV and ECD Spectra of Rhabdastrelloside A (**1**) in EtOH
S13 (−)HRESIMS of Rhabdastrelloside B (**2**) and MS/MS spectrum of its $[M - H]^-$ precursor ion at *m/z* 822
S14 HRESIMS (Positive Ion Mode) of Rhabdastrelloside B (**2**)
S15 ^{1}H NMR Spectrum of Rhabdastrelloside B (**2**) in CD_3OD (500 MHz)
S16 ^{13}C NMR Spectrum of Rhabdastrelloside B (**2**) in CD_3OD (500 MHz)
S17 HSQC Spectrum of Rhabdastrelloside B (**2**) in CD_3OD (500 MHz)
S18 HMBC Spectrum of Rhabdastrelloside B (**2**) in CD_3OD (700 MHz)
S19 COSY Spectrum of Rhabdastrelloside B (**2**) in CD_3OD (500 MHz)
S20 ROESY Spectrum of Rhabdastrelloside B (**2**) in CD_3OD (500 MHz)
S21 1D TOCSY Spectra of Rhabdastrelloside B (**2**) in CD_3OD (500 MHz)
S22 UV and ECD Spectra of Rhabdastrelloside B (**2**) in EtOH
S23 Determination of Absolute Configurations of Monosaccharides: GC-MS profiles.
S24 Determination of Absolute Configurations of Monosaccharides: GC-MS profiles for sum of Standard L-Glc and D-Glc, and Standard L-2-NHAcGlc.
S25 Determination of Absolute Configurations of Monosaccharides: EIMS spectra of detected D-Glc and Standard D-Glc.
S26 Determination of Absolute Configurations of Monosaccharides: EIMS spectra of detected D-Glc and Standard D-Glc (Continuation).
S27 Determination of Absolute Configurations of Monosaccharides: EIMS spectra of detected D-2-NHAcGlc and Standard D-2-NHAcGlc.
S28 The Isolation Scheme

S1 (−)HRESIMS of Rhabdastrelloside A (**1**) and MS/MS spectrum of its [M − H]$^-$ precursor ion at *m/z* 838

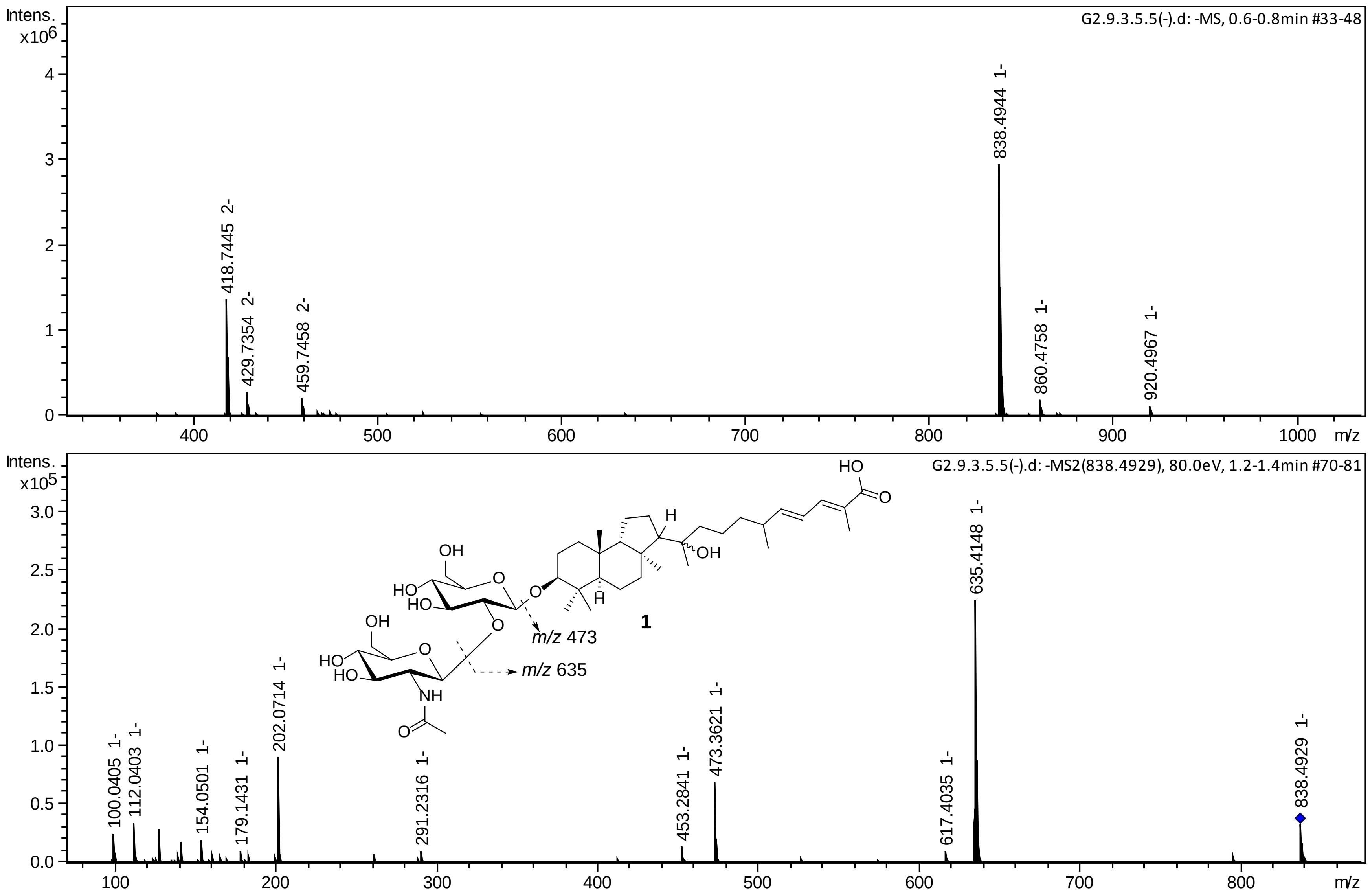

S2 HRESIMS (Positive Ion Mode) of Rhabdastrelloside A (**1**)

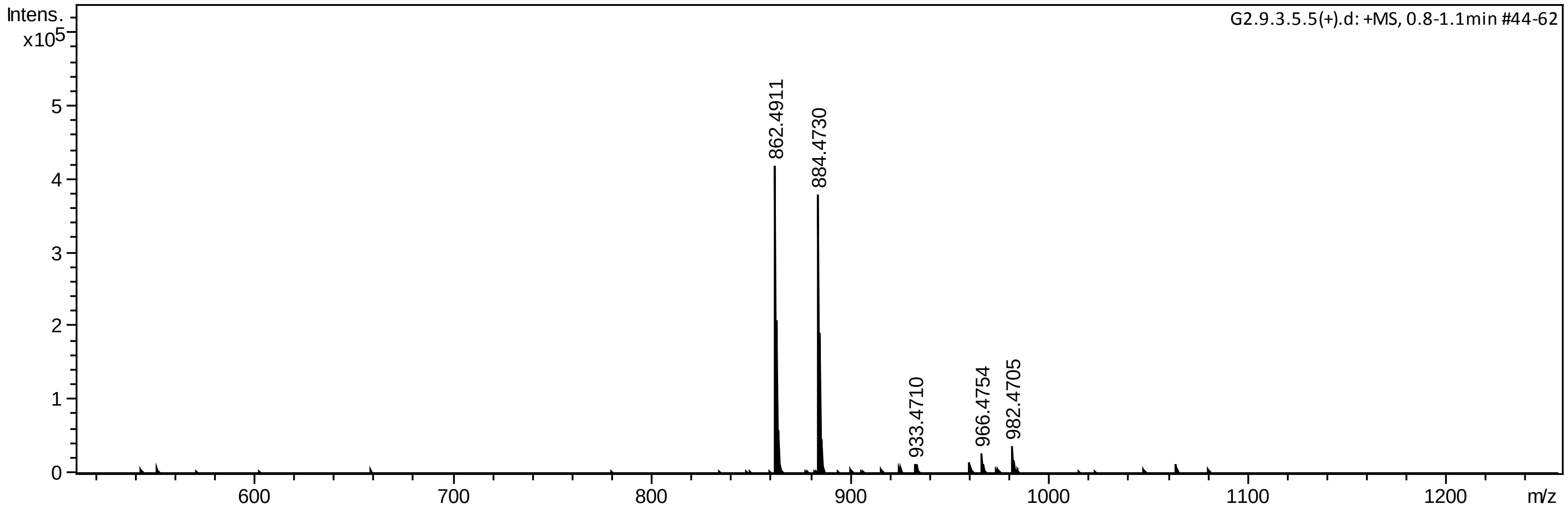

S3 ^{1}H NMR Spectrum of Rhabdastrelloside A (**1**) in CD_3OD (700 MHz)

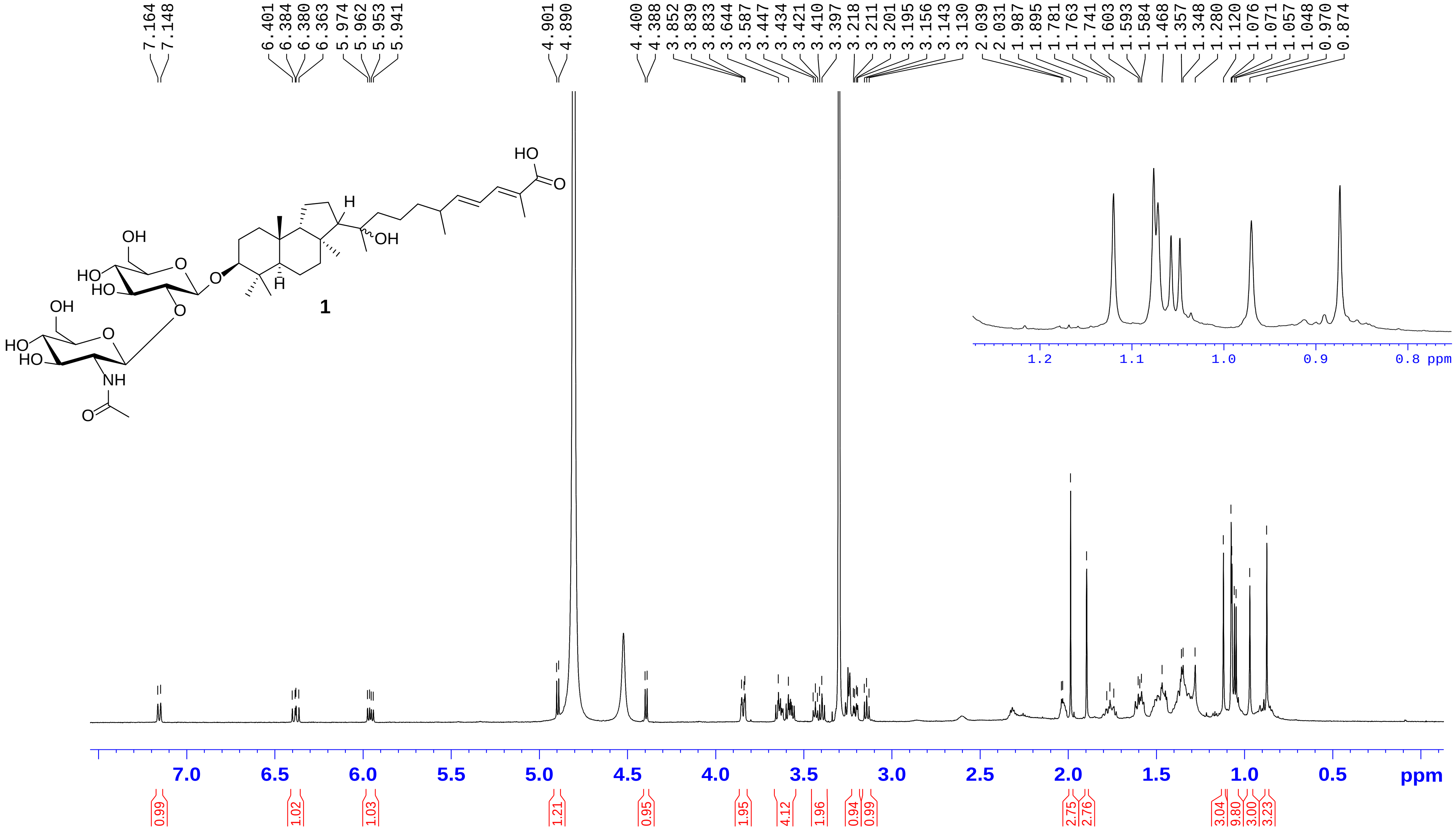

S4 ^{13}C NMR Spectrum of Rhabdastrelloside A (**1**) in CD_3OD (700 MHz)

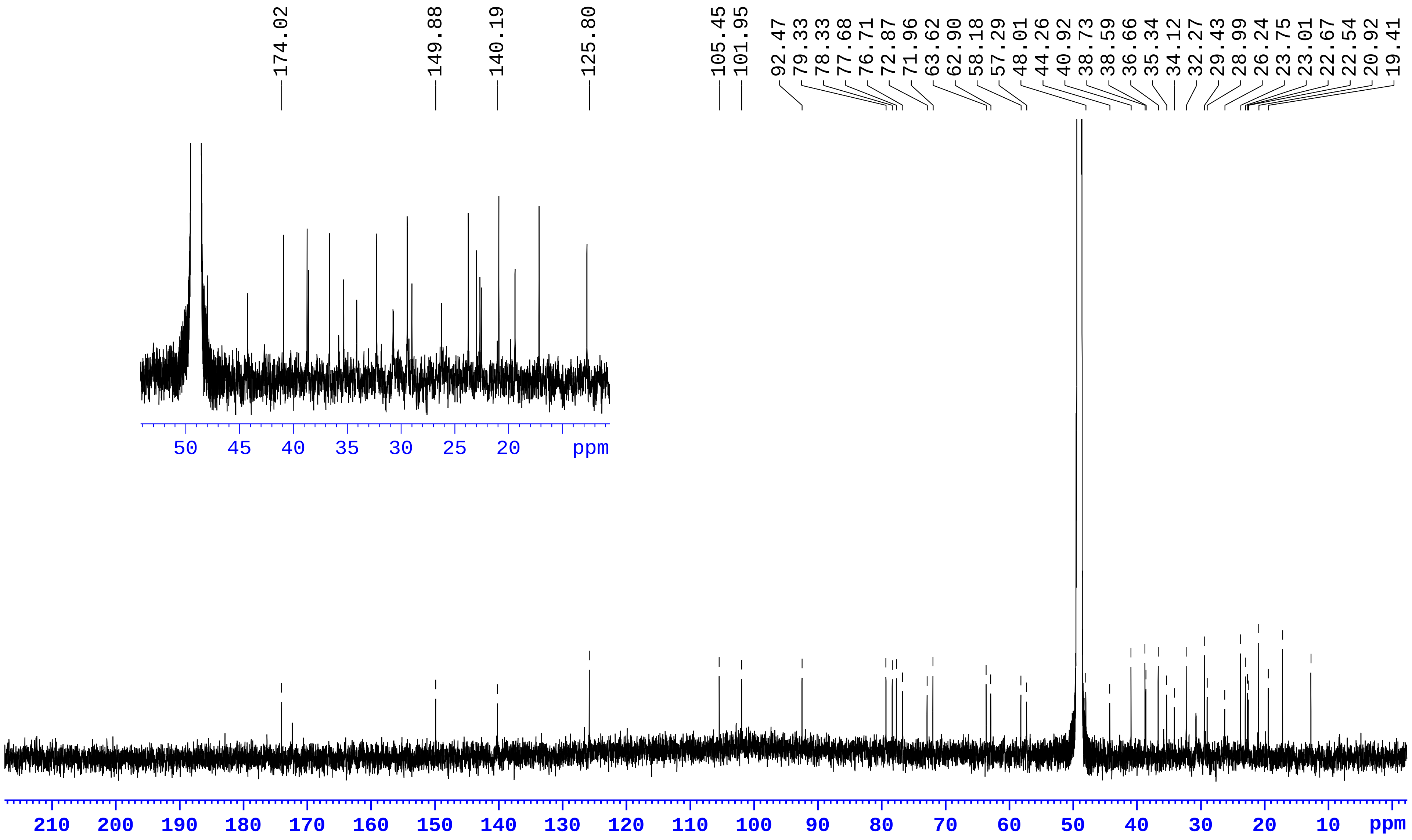

S5 HSQC Spectrum of Rhabdastrelloside A (**1**) in CD_3OD (700 MHz)

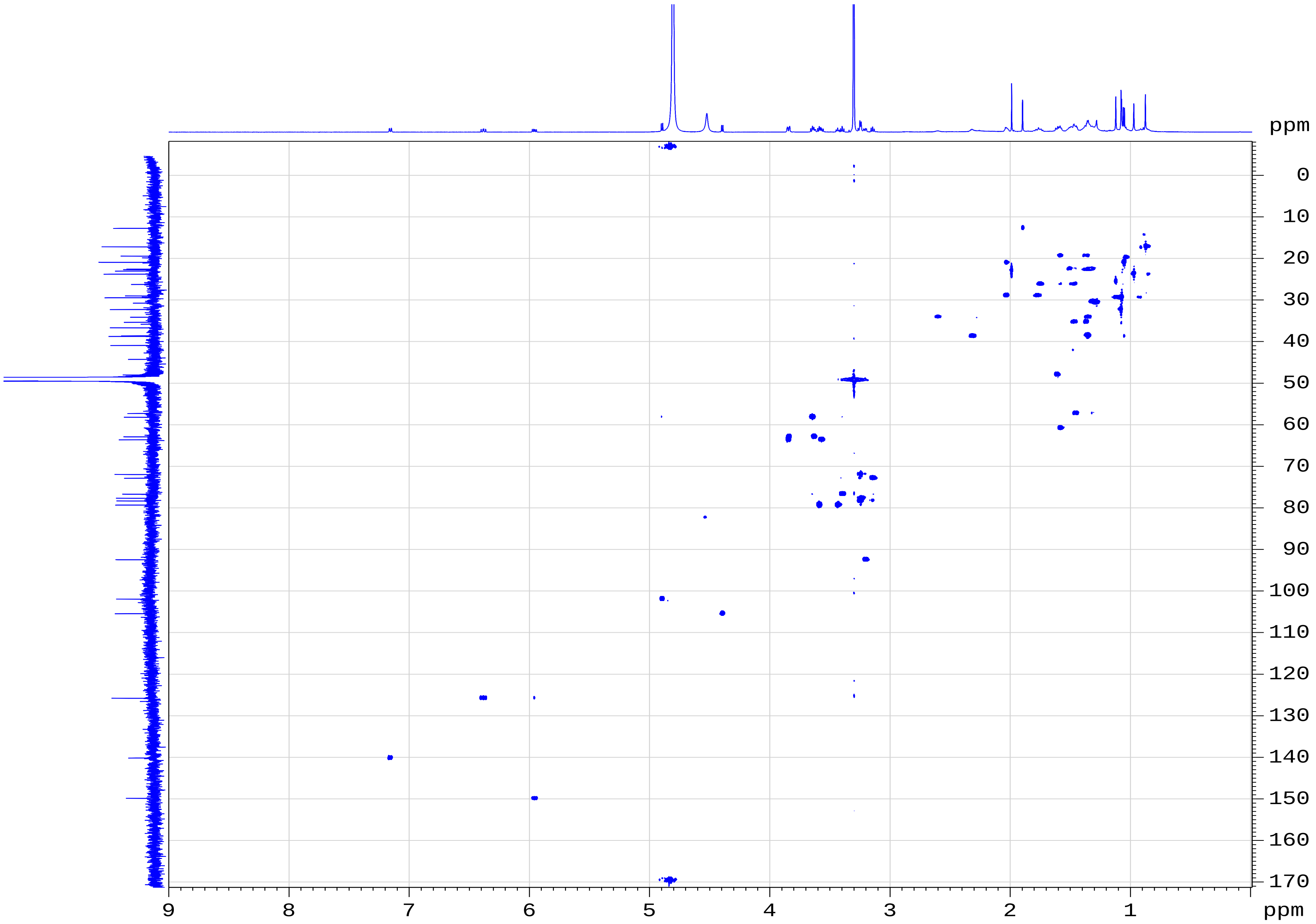

S6 HMBC Spectrum of Rhabdastrelloside A (**1**) in CD_3OD (700 MHz)

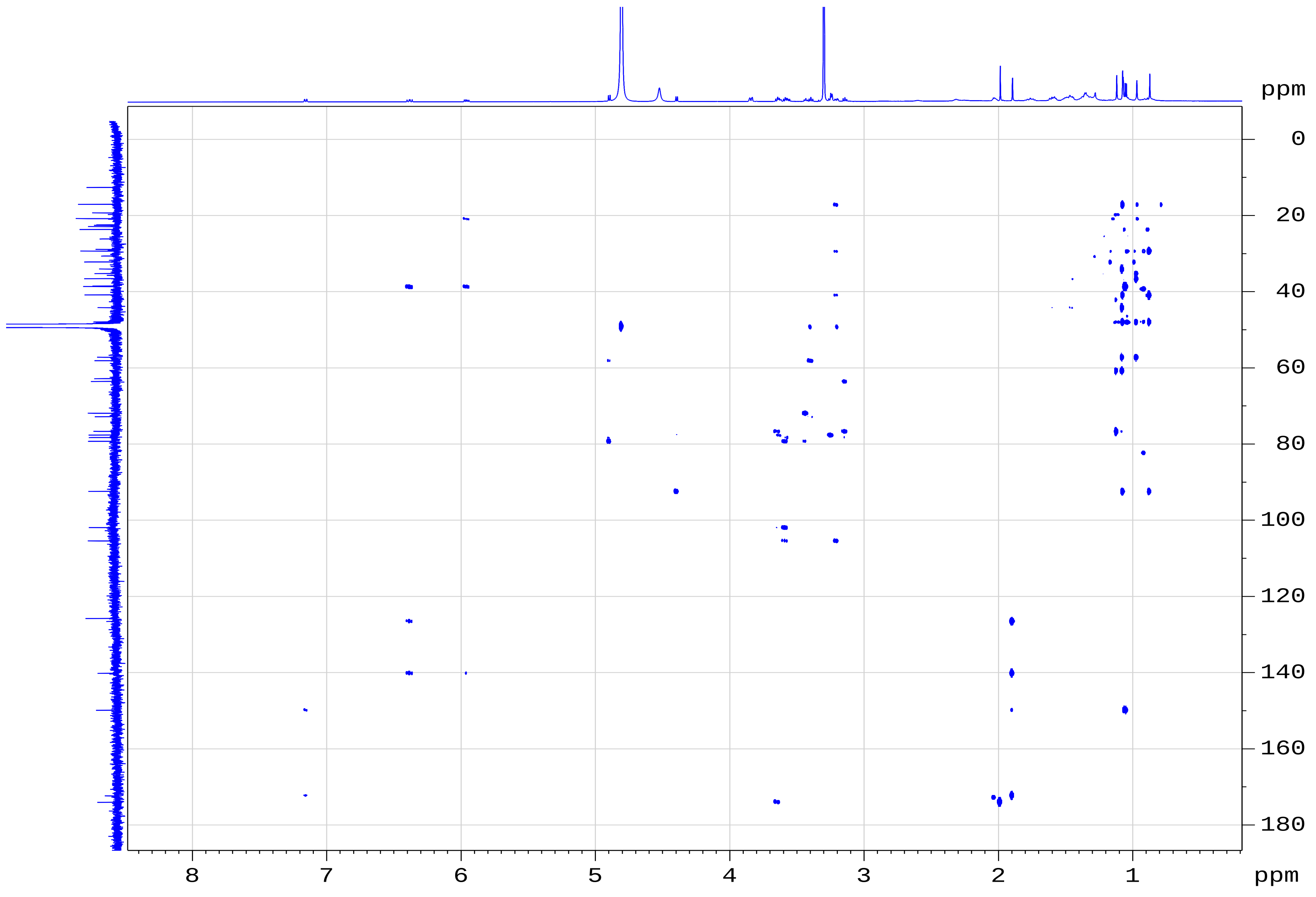

S7 COSY Spectrum of Rhabdastrelloside A (**1**) in CD_3OD (700 MHz)

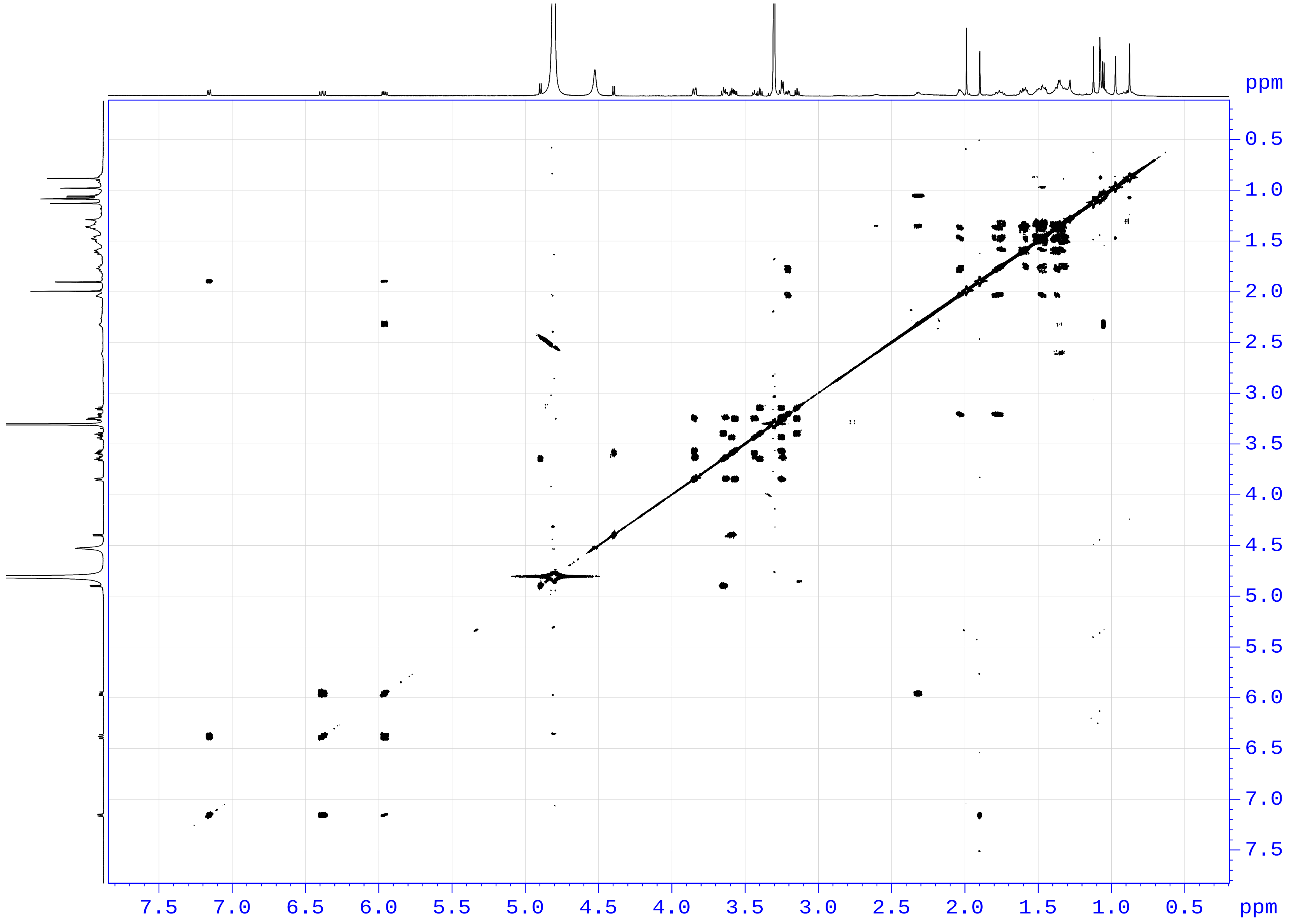

S8 Expanded COSY Spectrum of Rhabdastrelloside A (**1**) in CD_3OD (700 MHz)

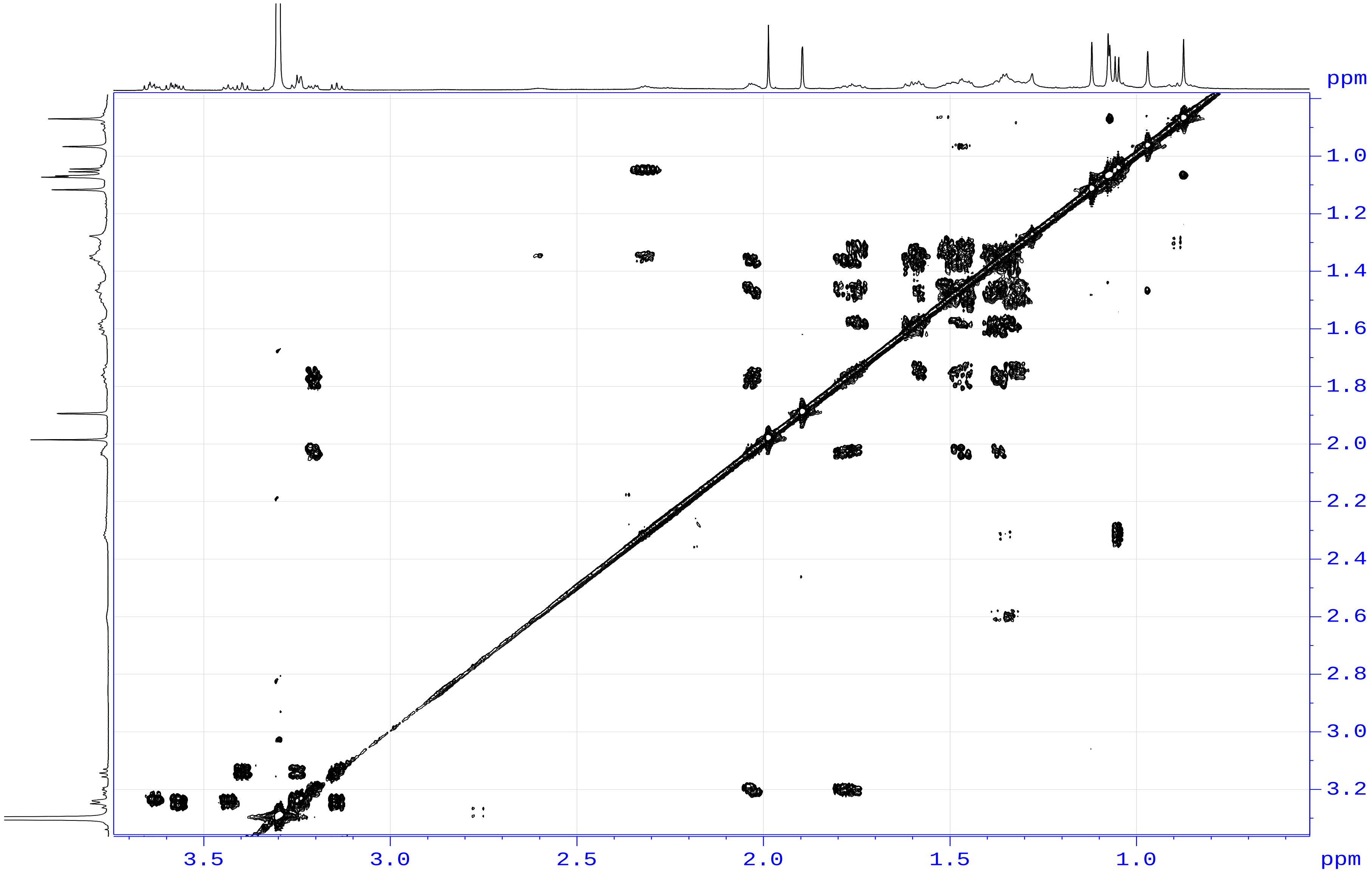

S9 2D TOCSY Spectrum of Rhabdastrelloside A (**1**) in CD_3OD (700 MHz)

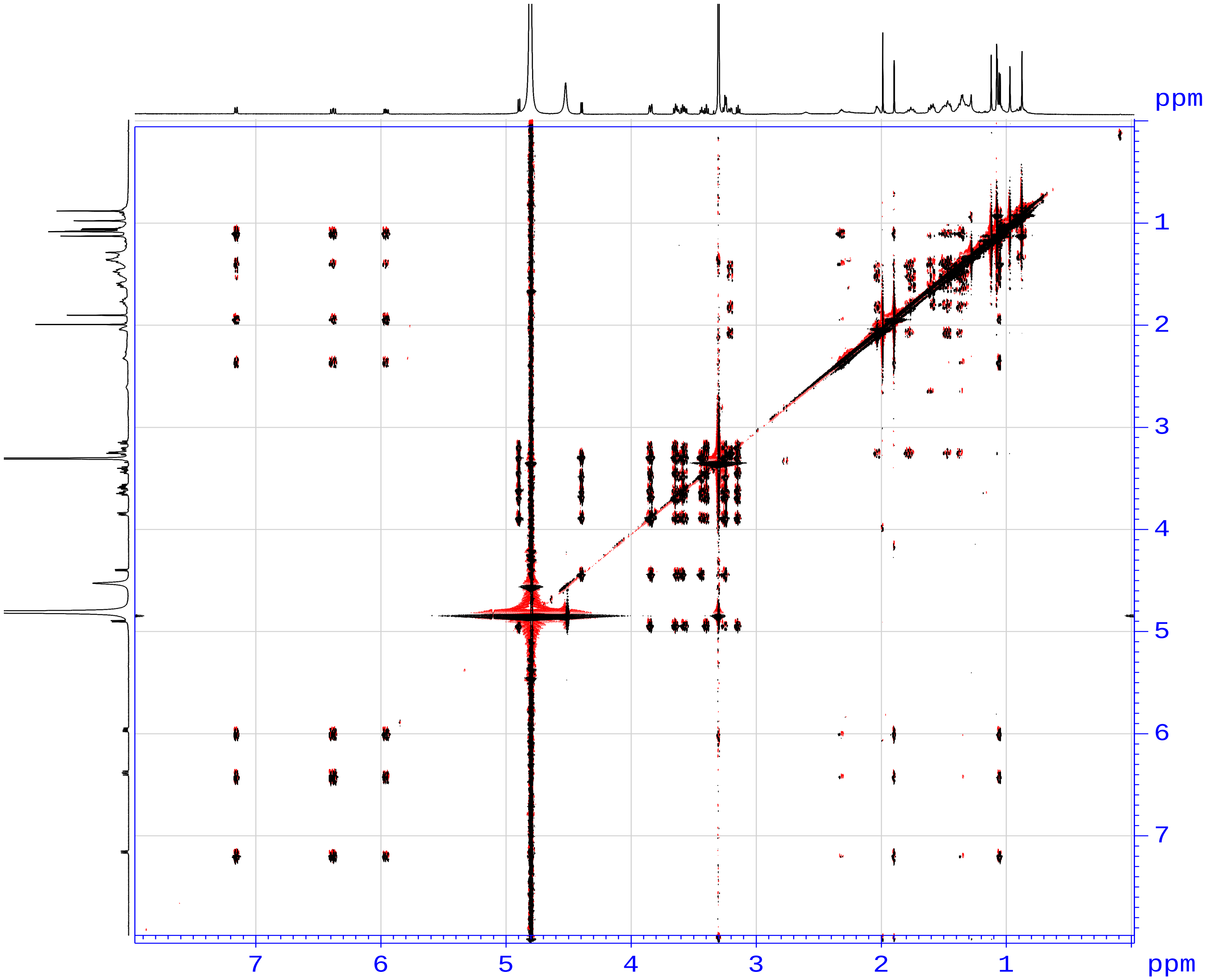

S10 Expanded 2D TOCSY Spectrum for disaccharide moiety in Rhabdastrelloside A (**1**) in CD_3OD (700 MHz)

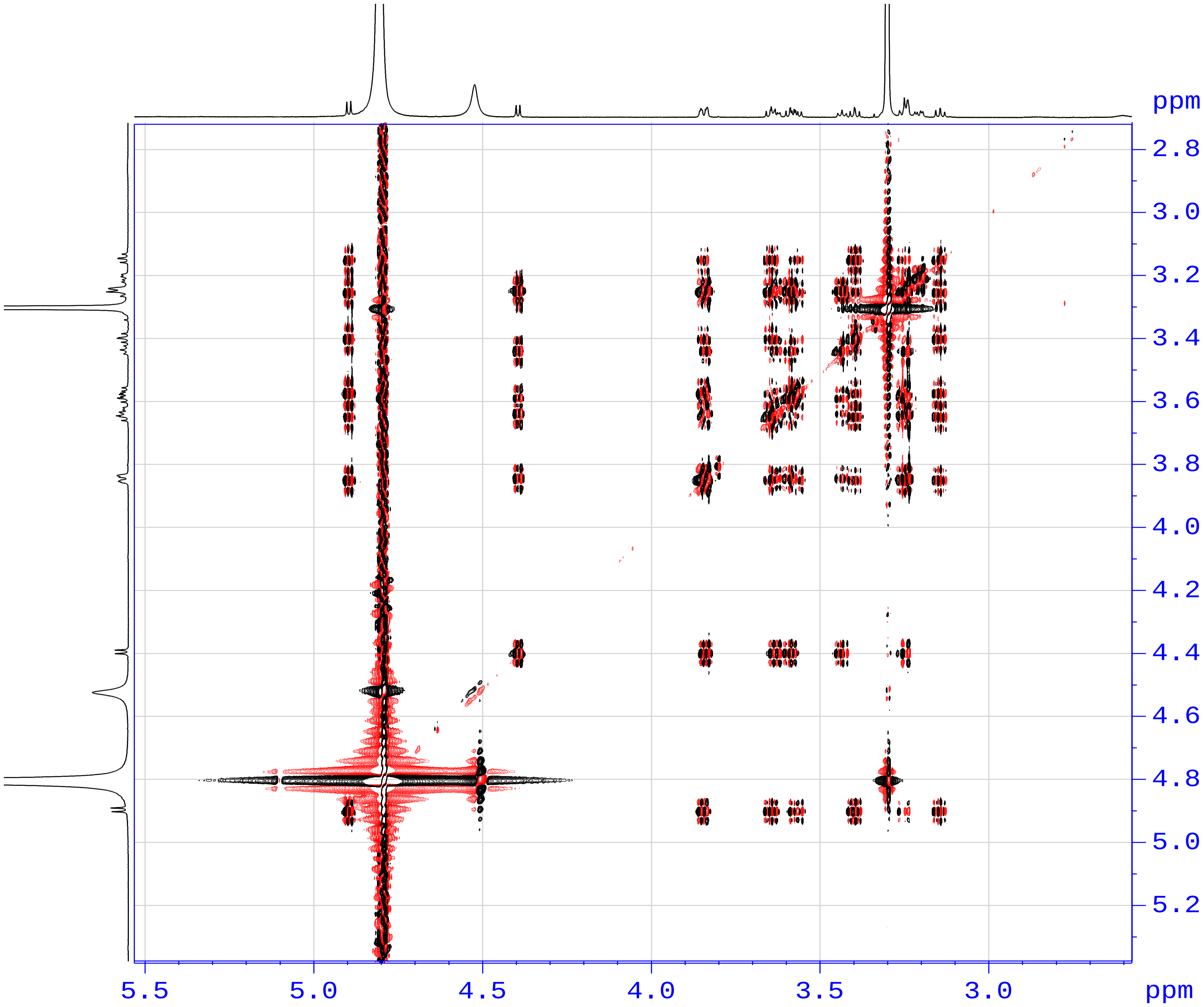

S11 ROESY Spectrum of Rhabdastrelloside A (**1**) in CD_3OD (700 MHz)

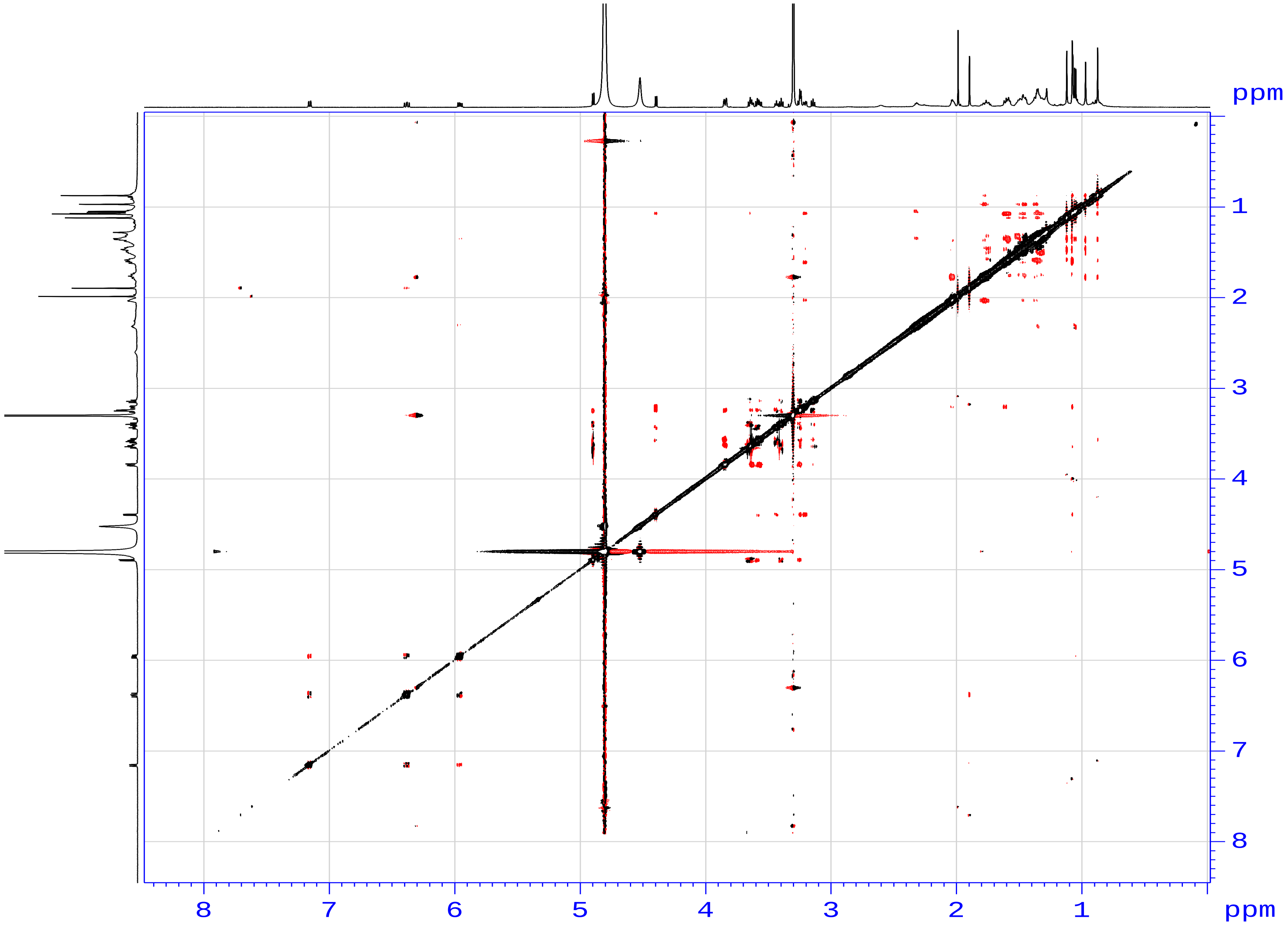

S12 UV and ECD Spectra of Rhabdastrelloside A (**1**) in EtOH

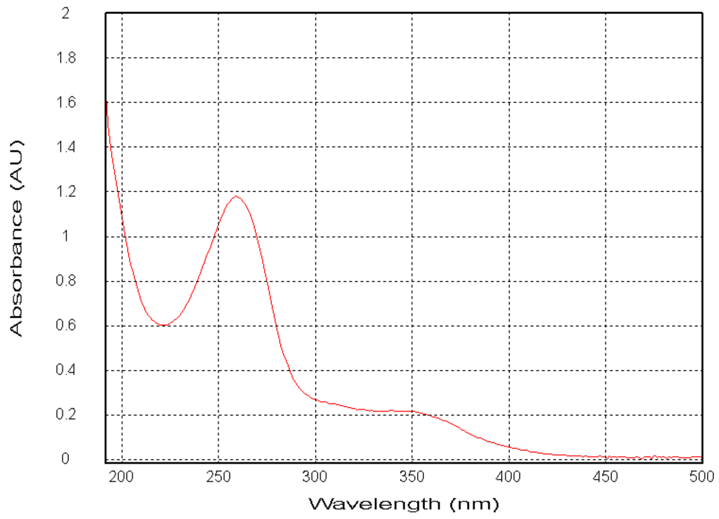

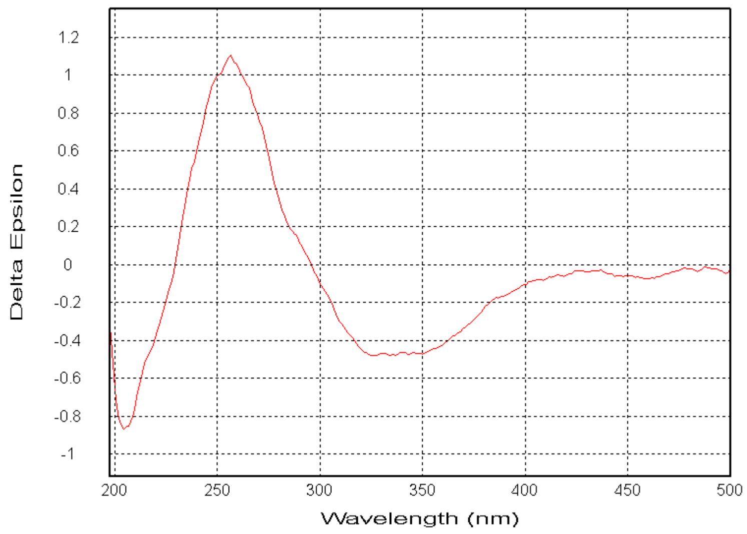

S13 (−)HRESIMS of Rhabdastrelloside B (**2**) and MS/MS spectrum of its [M − H]⁻ precursor ion at *m/z* 822

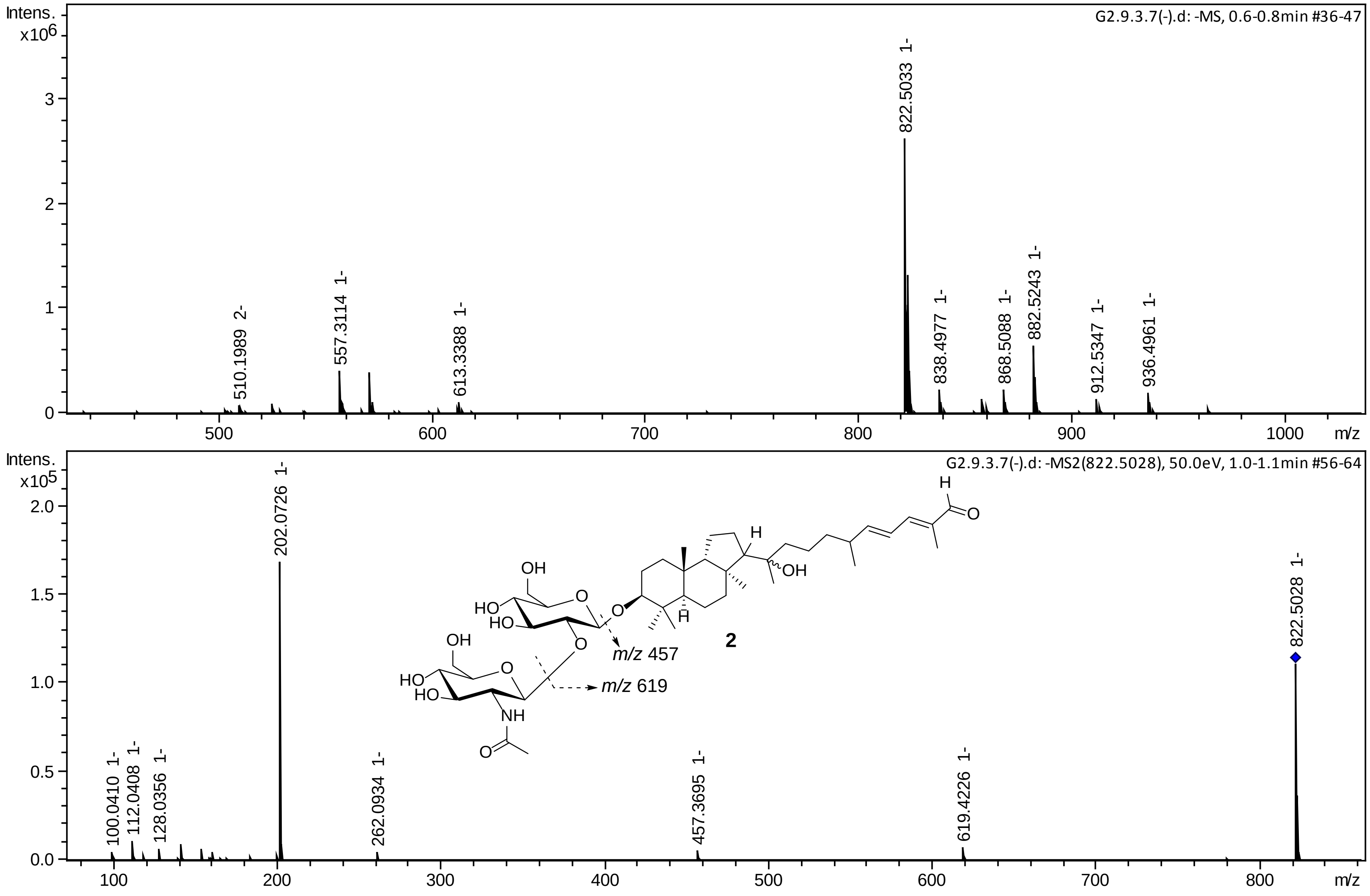

S14 HRESIMS (Positive Ion Mode) of Rhabdastrelloside B (**2**)

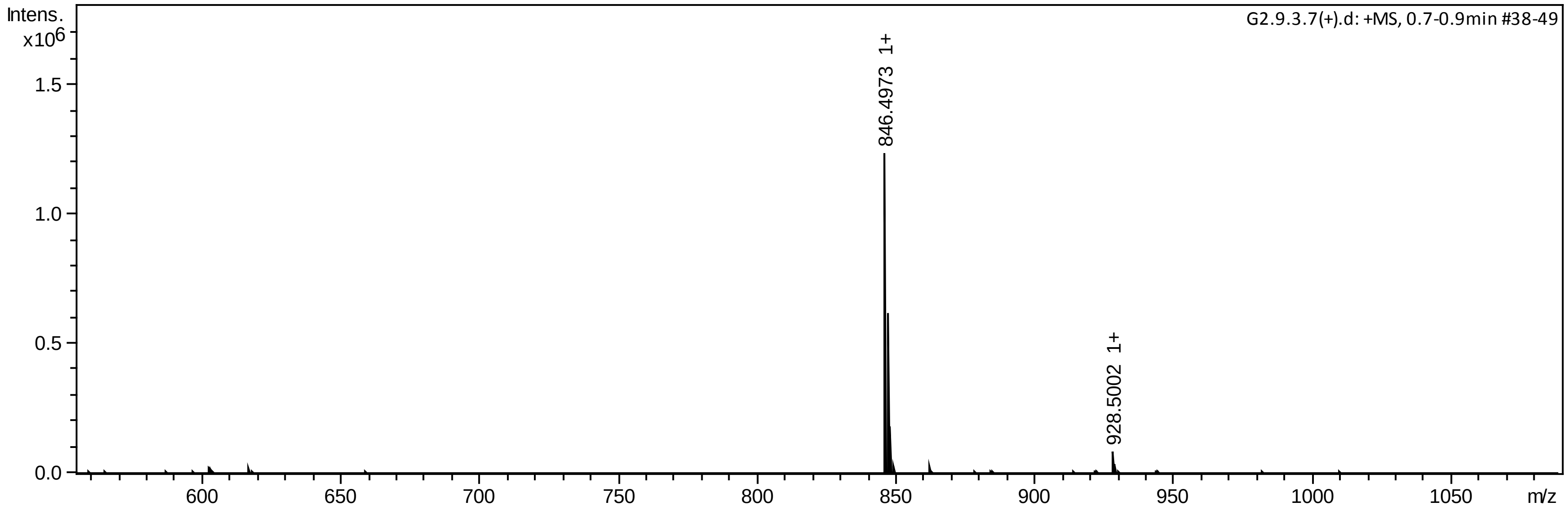

S15 ^{1}H NMR Spectrum of Rhabdastrelloside B (**2**) in CD_3OD (500 MHz)

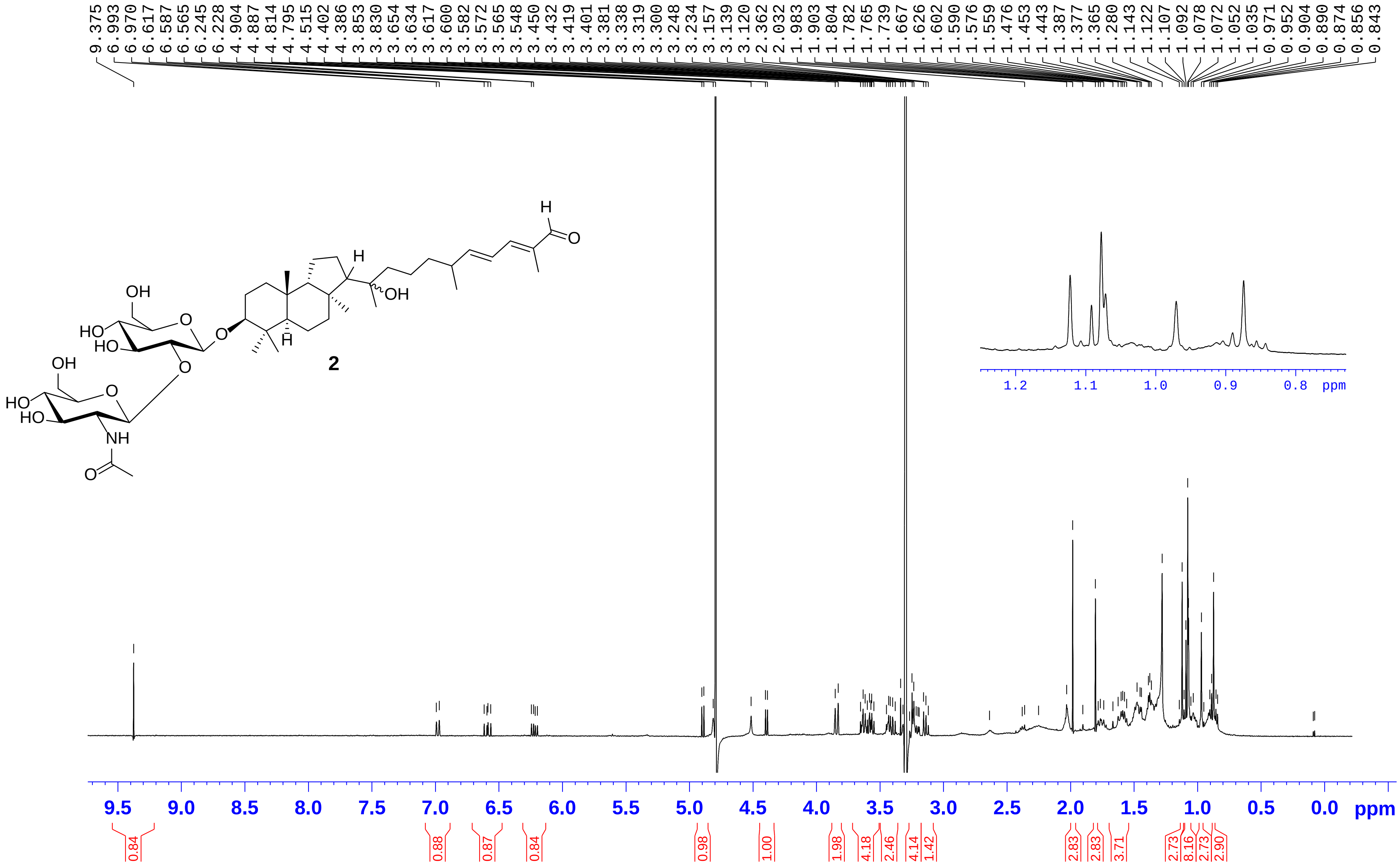

S16 ^{13}C NMR Spectrum of Rhabdastrelloside B (**2**) in CD_3OD (500 MHz)

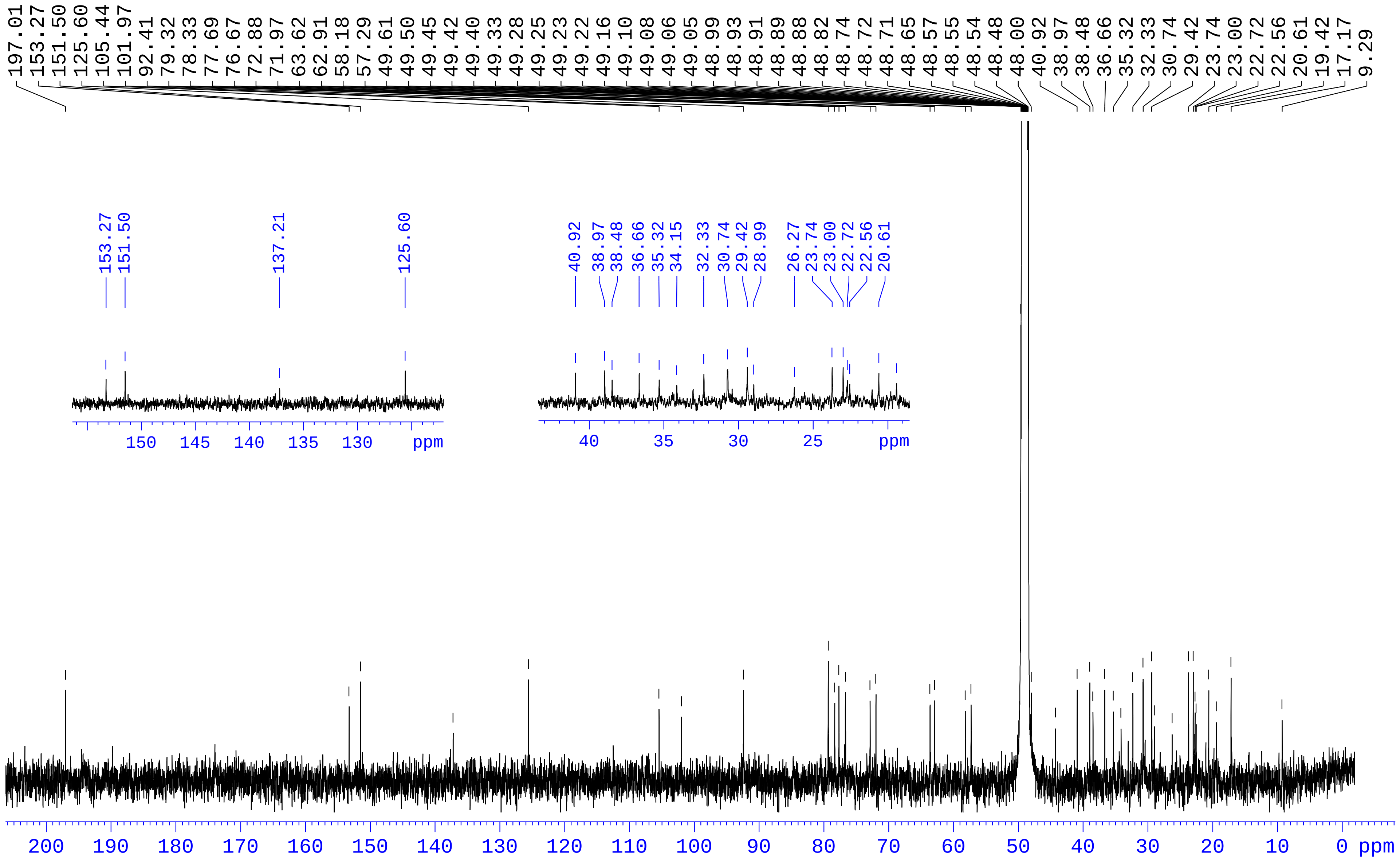

S17 HSQC Spectrum of Rhabdastrelloside B (**2**) in CD_3OD (500 MHz)

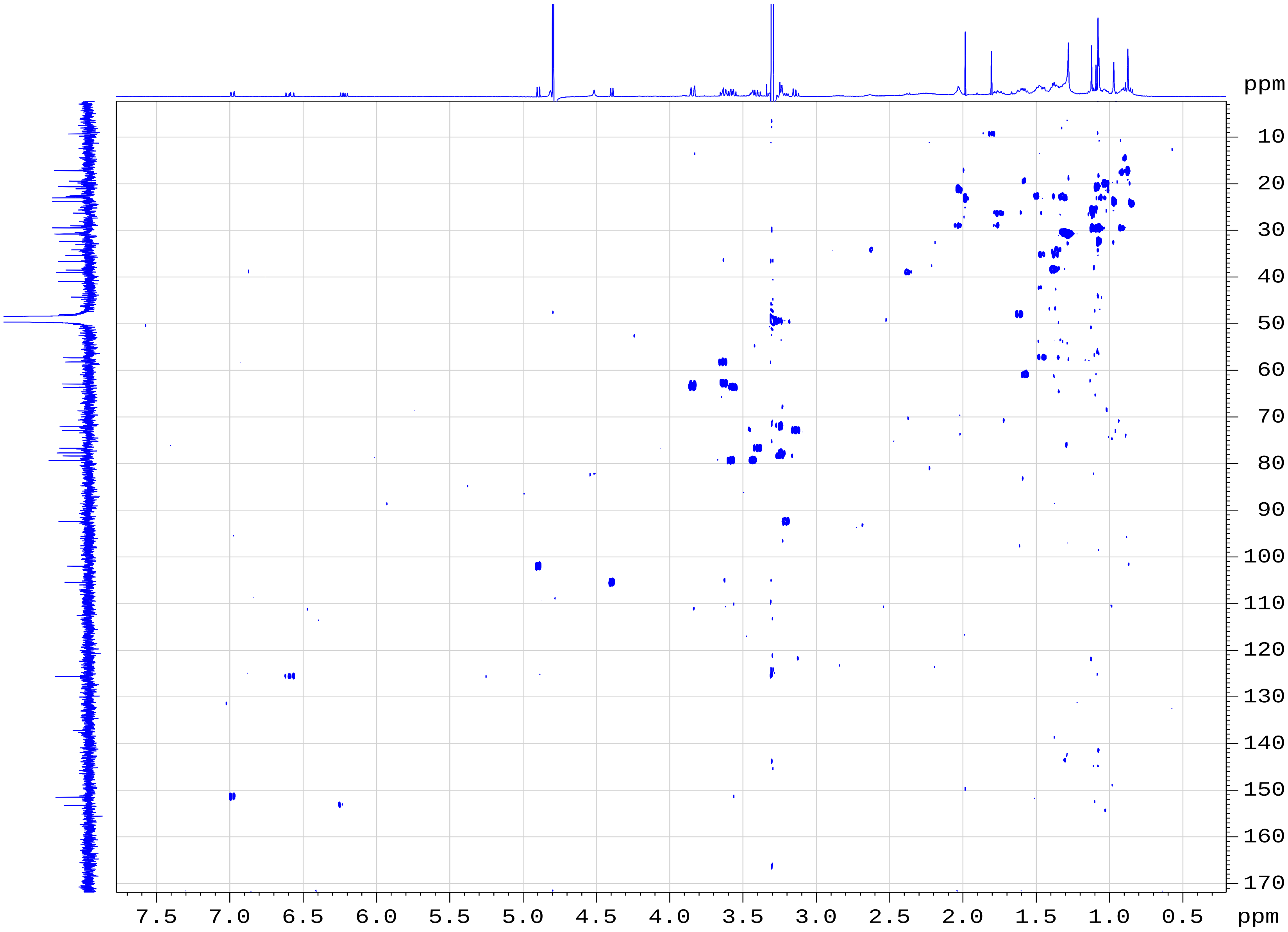

S18 HMBC Spectrum of Rhabdastrelloside B (**2**) in CD_3OD (700 MHz)

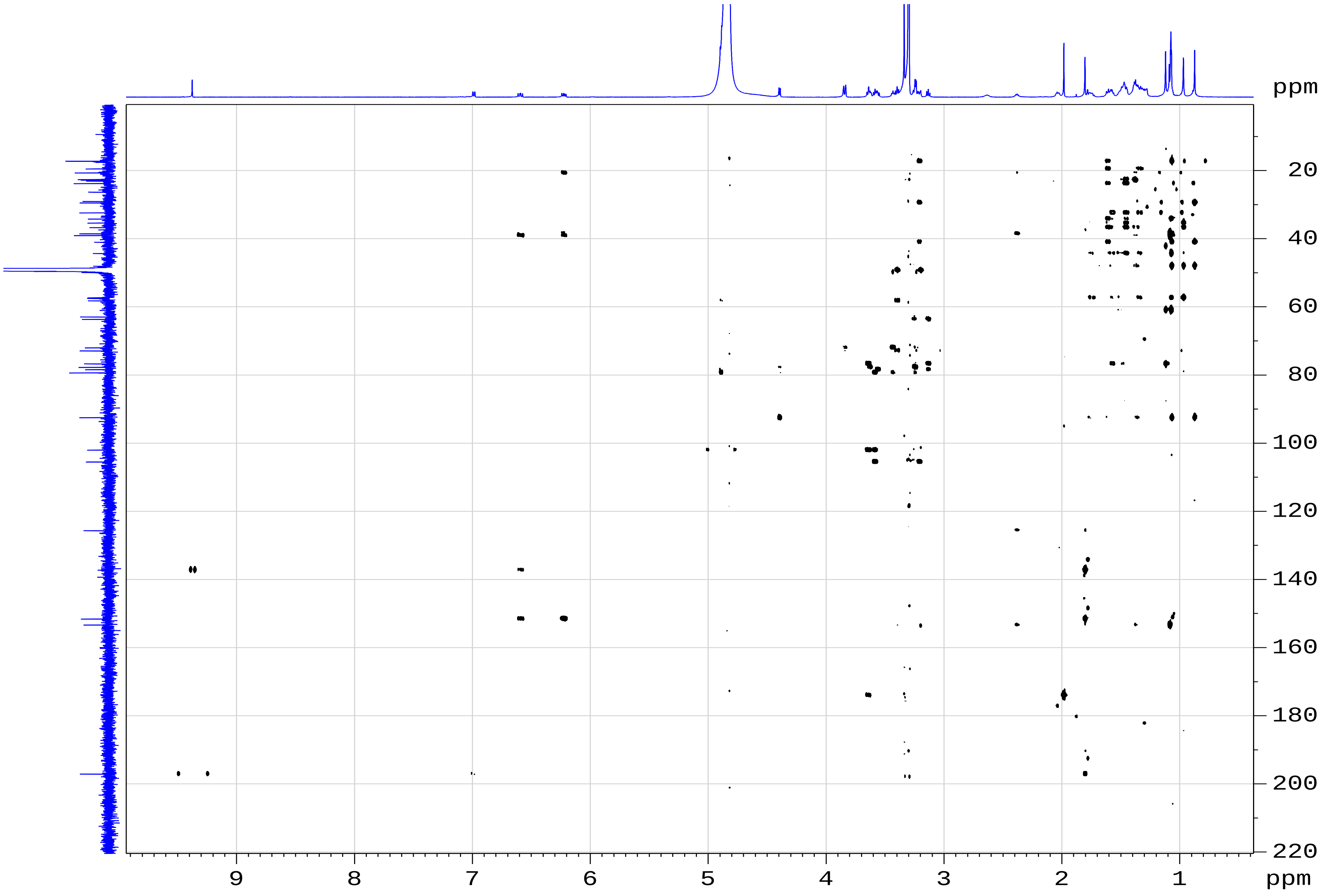

S19 COSY Spectrum of Rhabdastrelloside B (**2**) in CD_3OD (500 MHz)

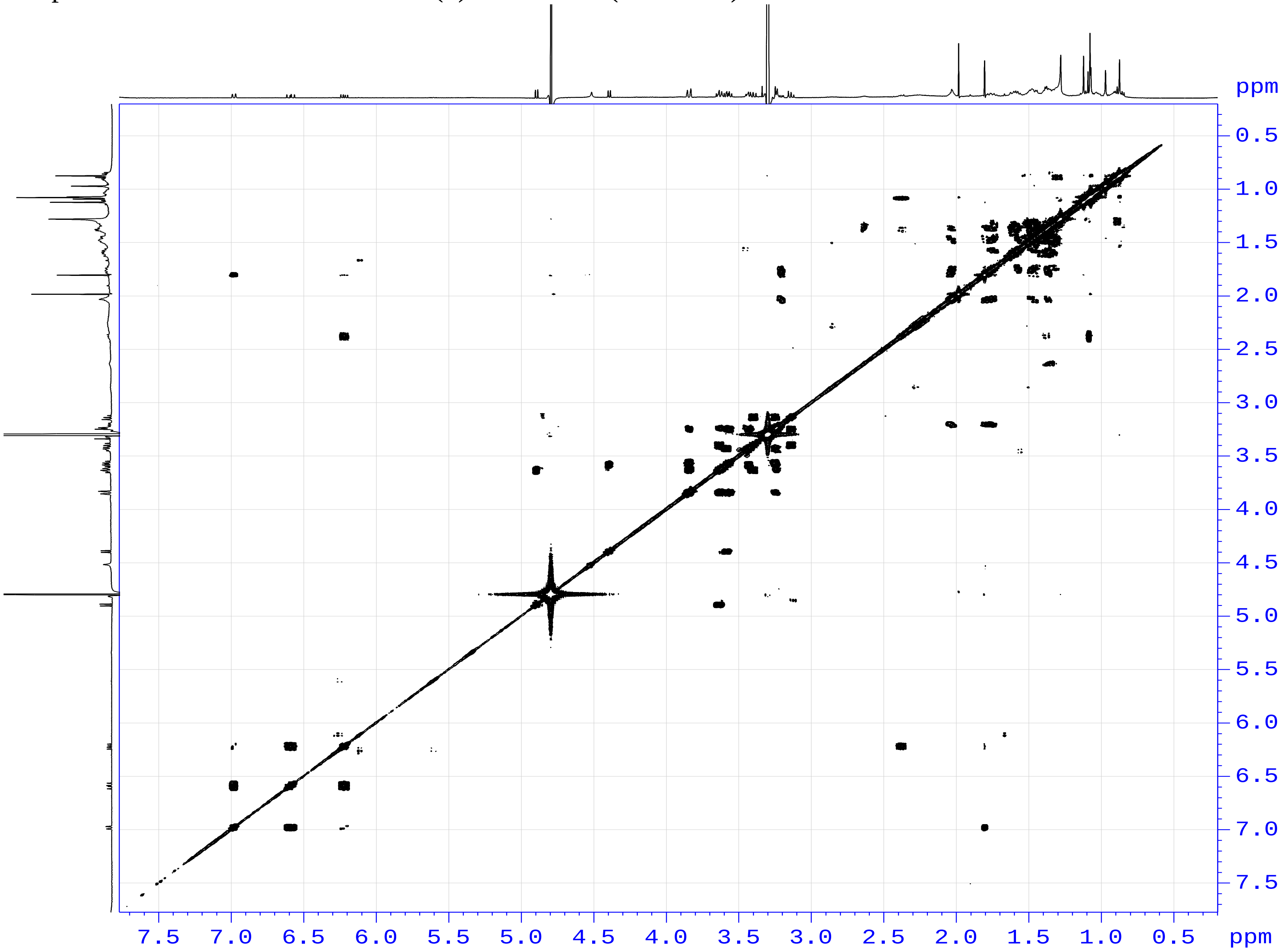

S20 ROESY Spectrum of Rhabdastrelloside B (**2**) in CD_3OD (500 MHz)

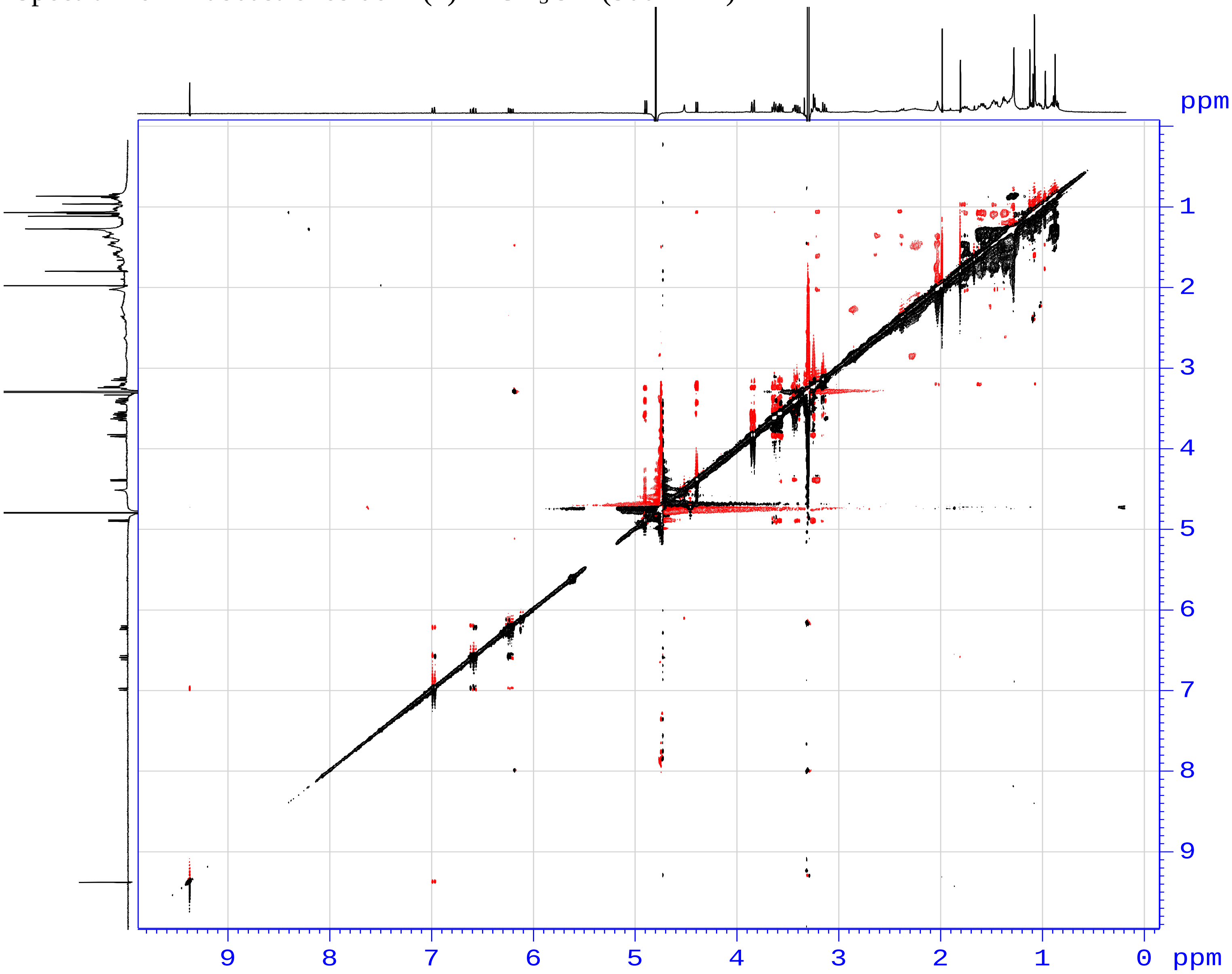

S21 1D TOCSY Spectra of Rhabdastrelloside B (**2**) in CD_3OD (500 MHz)

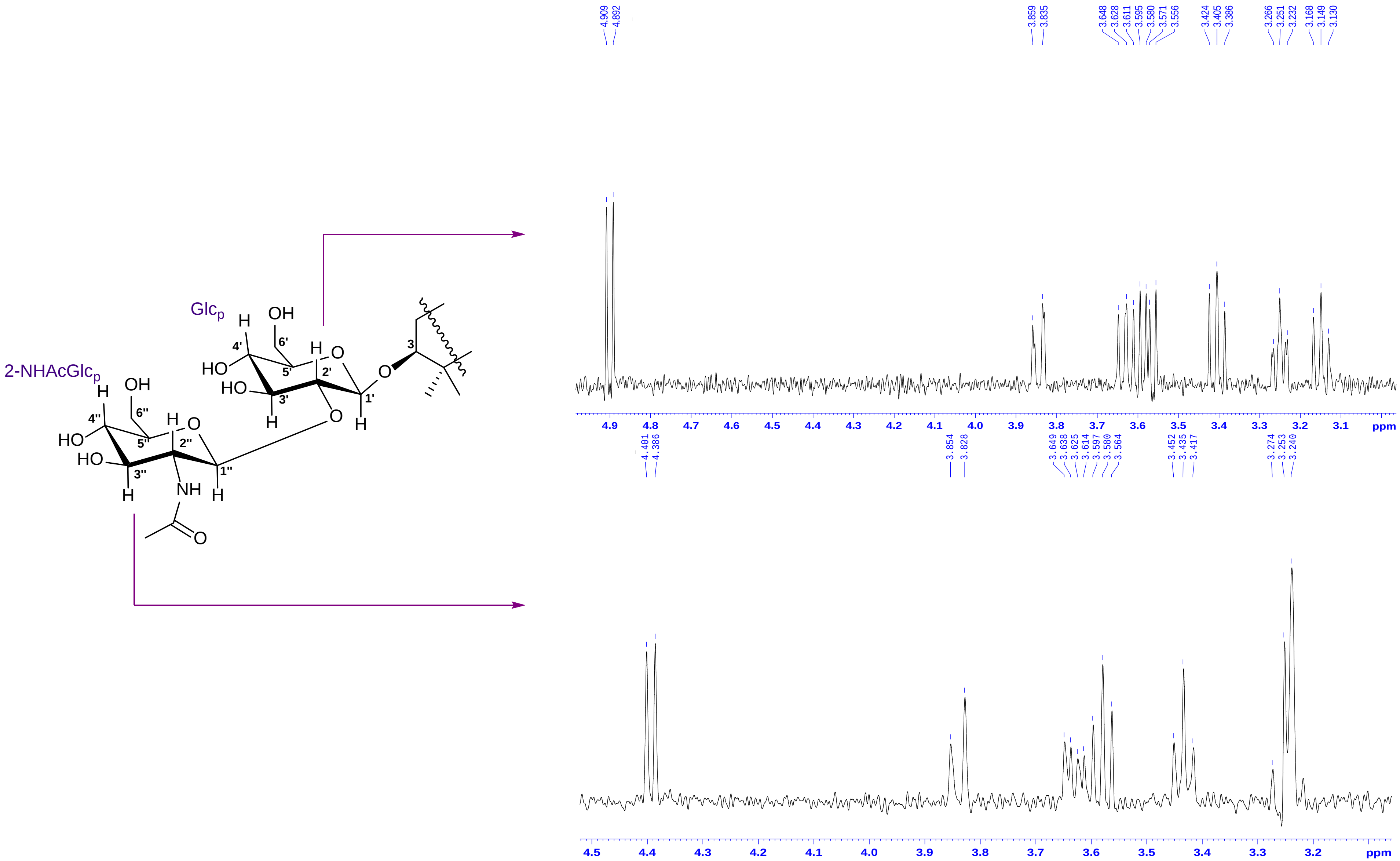

S22 UV and ECD Spectra of Rhabdastrelloside B (**2**) in EtOH

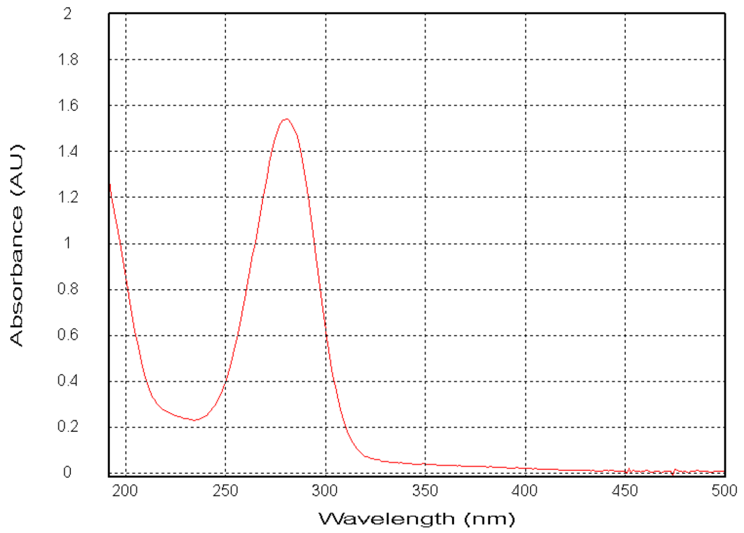

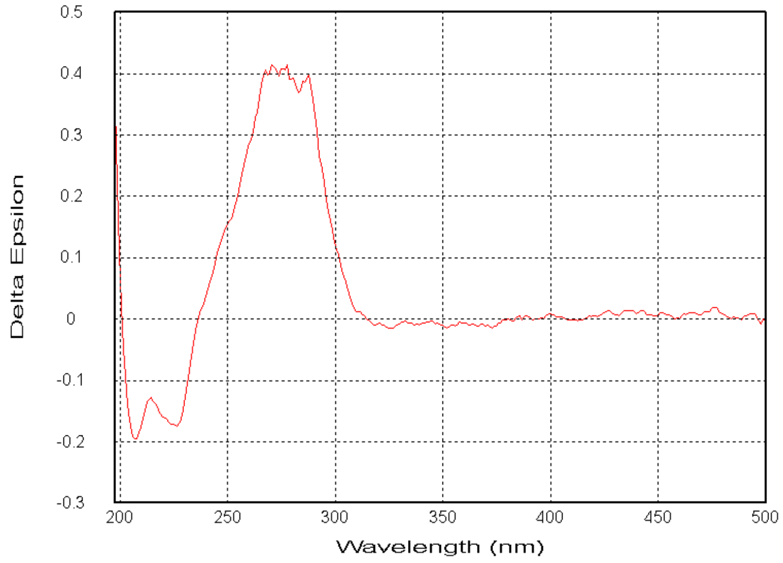

S23 Determination of Absolute Configurations of Monosaccharides: GC-MS profiles.

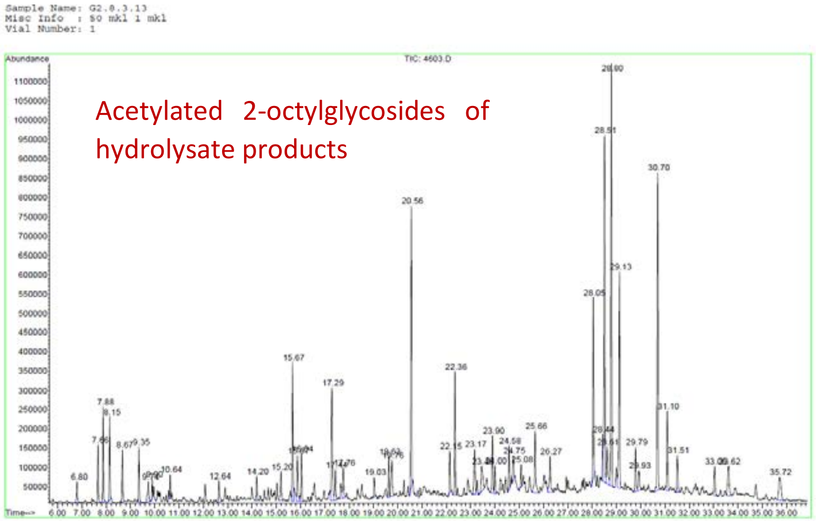

Acid hydrolysis of compound **2** (1.4 mg) was carried out as described in the text of the article (See part *4.5*) The acetylated 2-octylglycosides were analyzed by GC-MS using the corresponding authentic samples prepared by the same procedure.

GC-MS analyses were carried out on a Hewlett Packard HP6890 GC System, with a HP-5MS capillary column (30 m× 0.25 mm, 0.25 µm). The carrier gas was helium (flow rate 1.0 mL/min), the injector temperature was 270 °C, the ionizing voltage was 70 eV and the temperature program was 100 °C (0.5 min) −5 °C/min −250 °C (10 min).

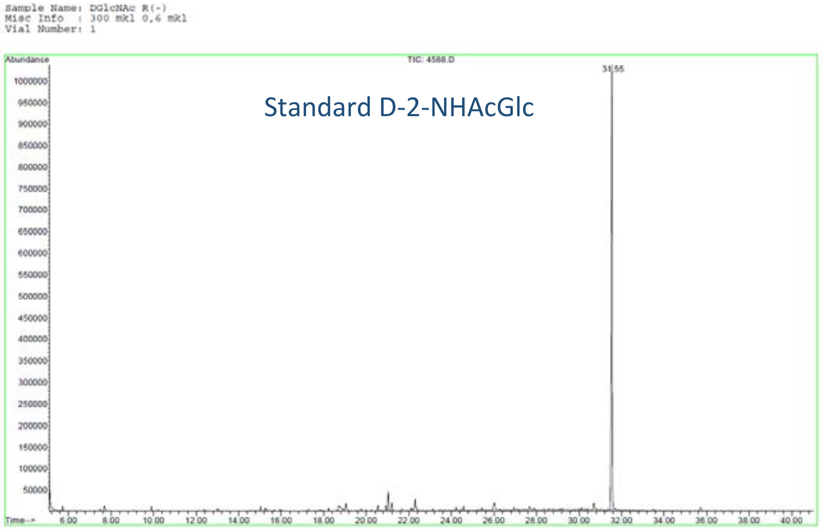

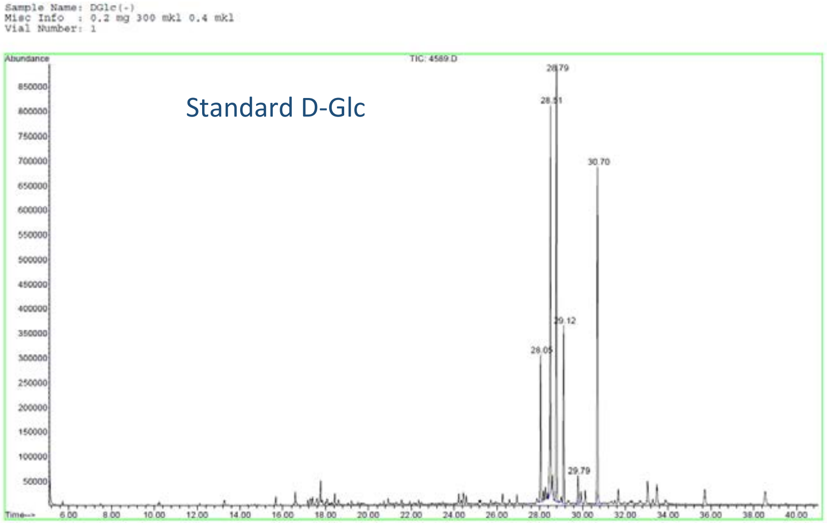

S24 Determination of Absolute Configurations of Monosaccharides: GC-MS profiles for sum of Standard L-Glc and D-Glc, and Standard L-2-NHAcGlc.

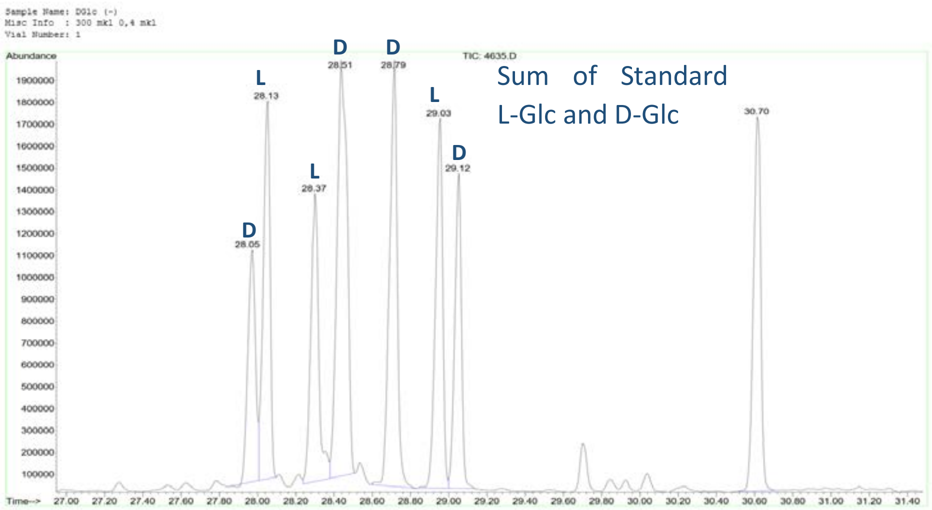

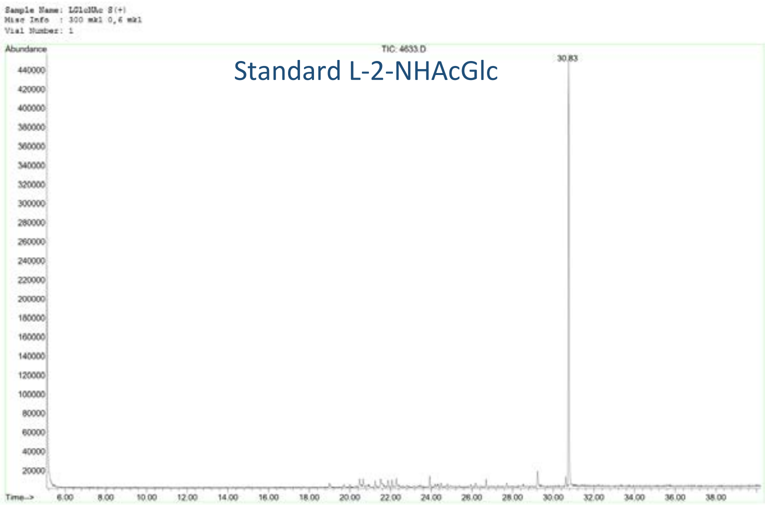

According to the retention times (t_R) and corresponding mass-spectra, L-monosaccharides were not found among acetylated 2-octylglycosides of hydrolysate products.

S25 Determination of Absolute Configurations of Monosaccharides: EIMS spectra of detected D-Glc and Standard D-Glc.

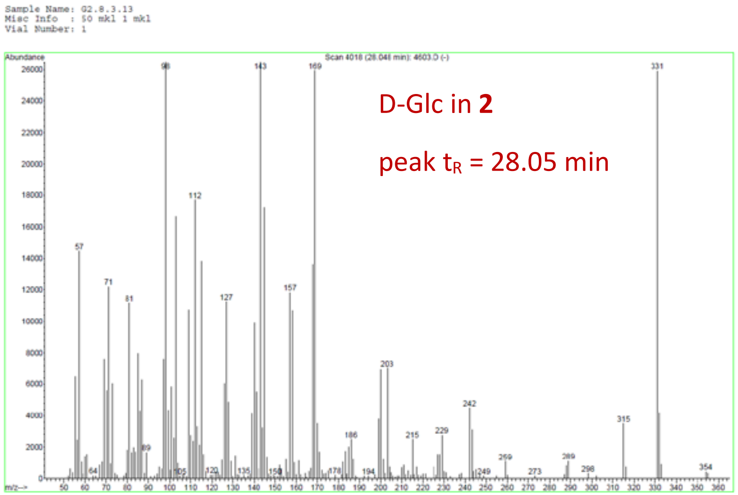

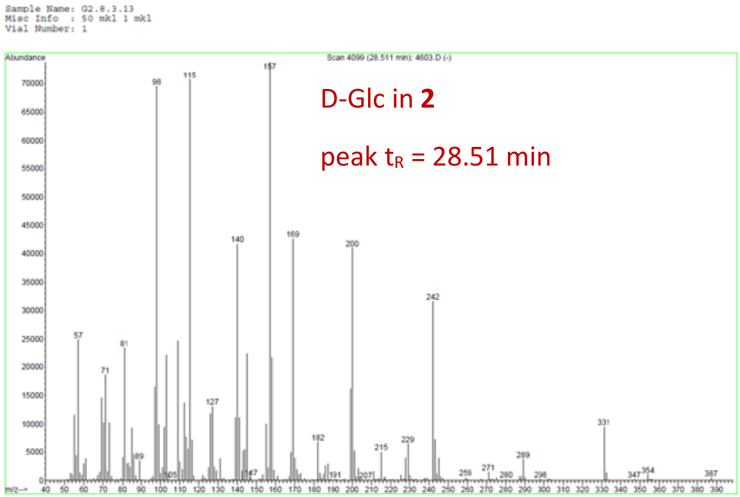

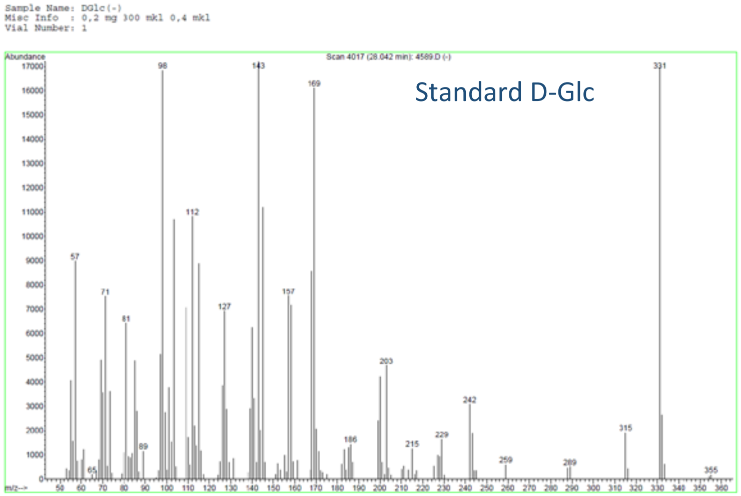

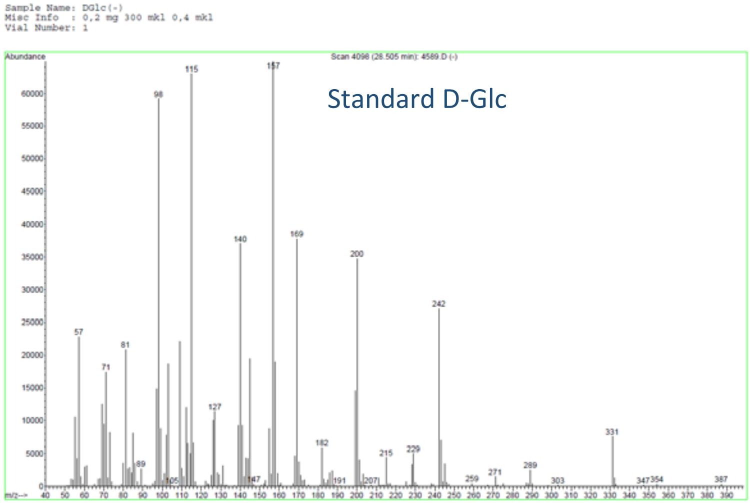

S26 Determination of Absolute Configurations of Monosaccharides: EIMS spectra of detected D-Glc and Standard D-Glc (Continuation).

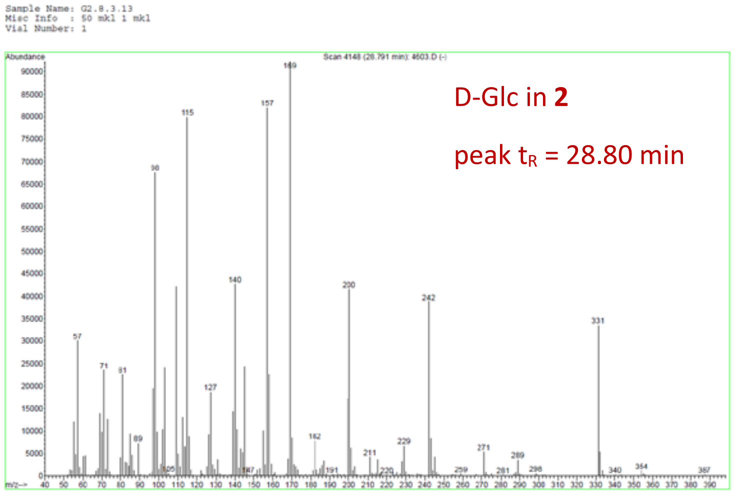

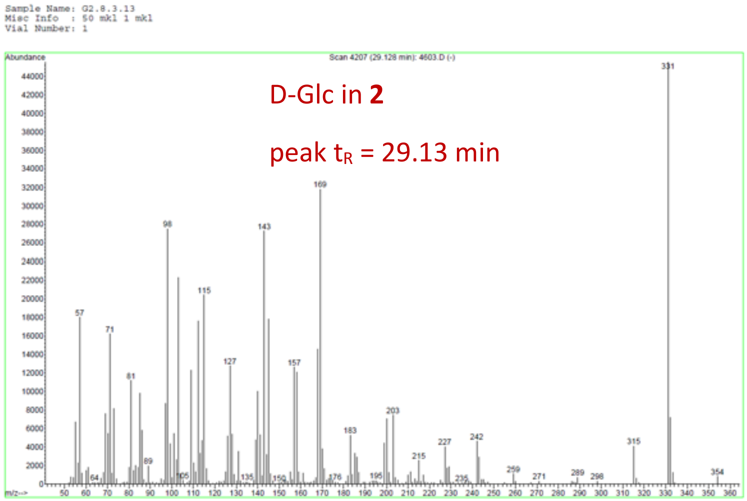

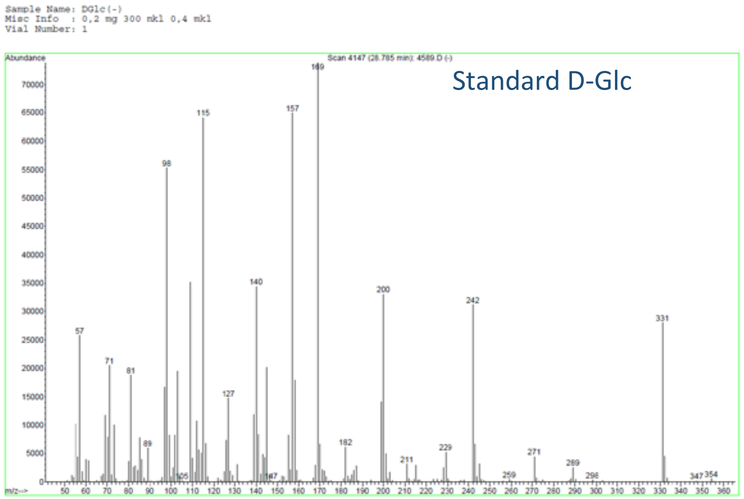

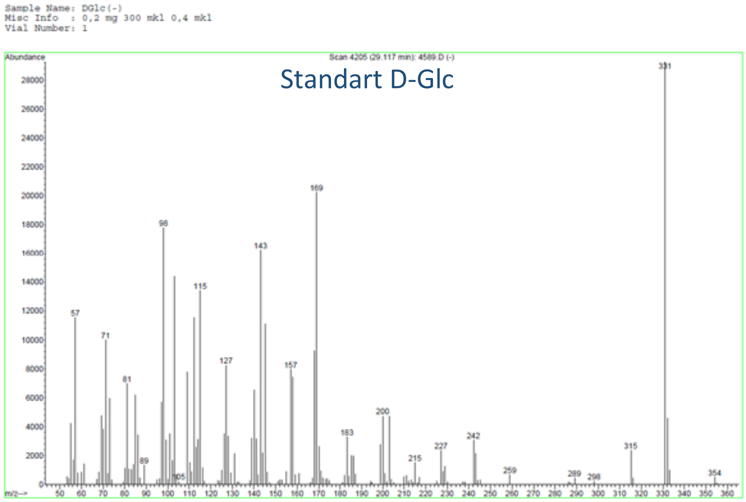

S27 Determination of Absolute Configurations of Monosaccharides: EIMS spectra of detected D-2-NHAcGlc and Standard D-2-NHAcGlc.

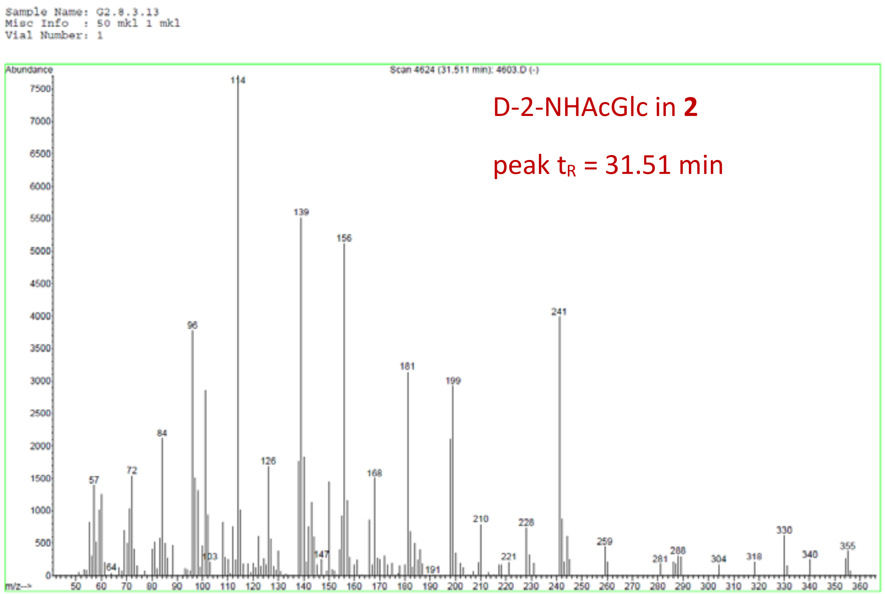

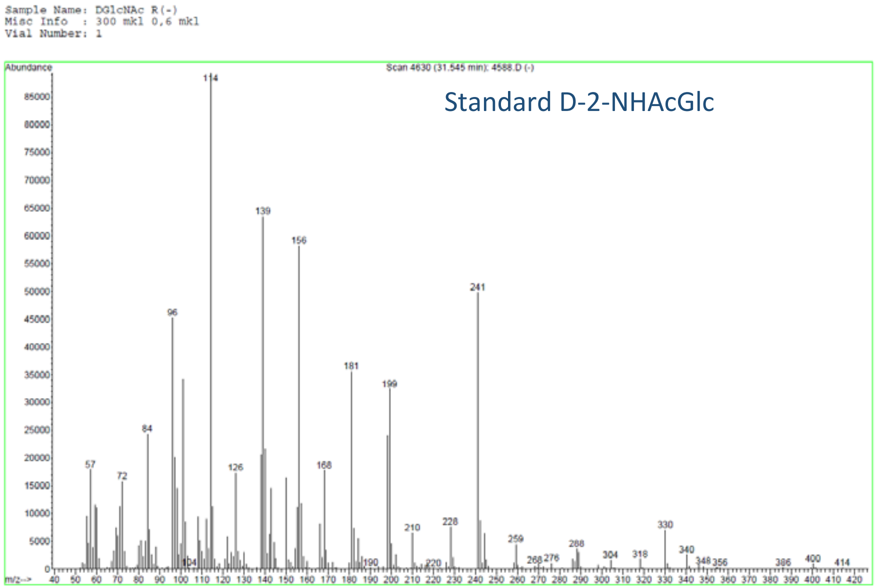

S28 The Isolation Scheme

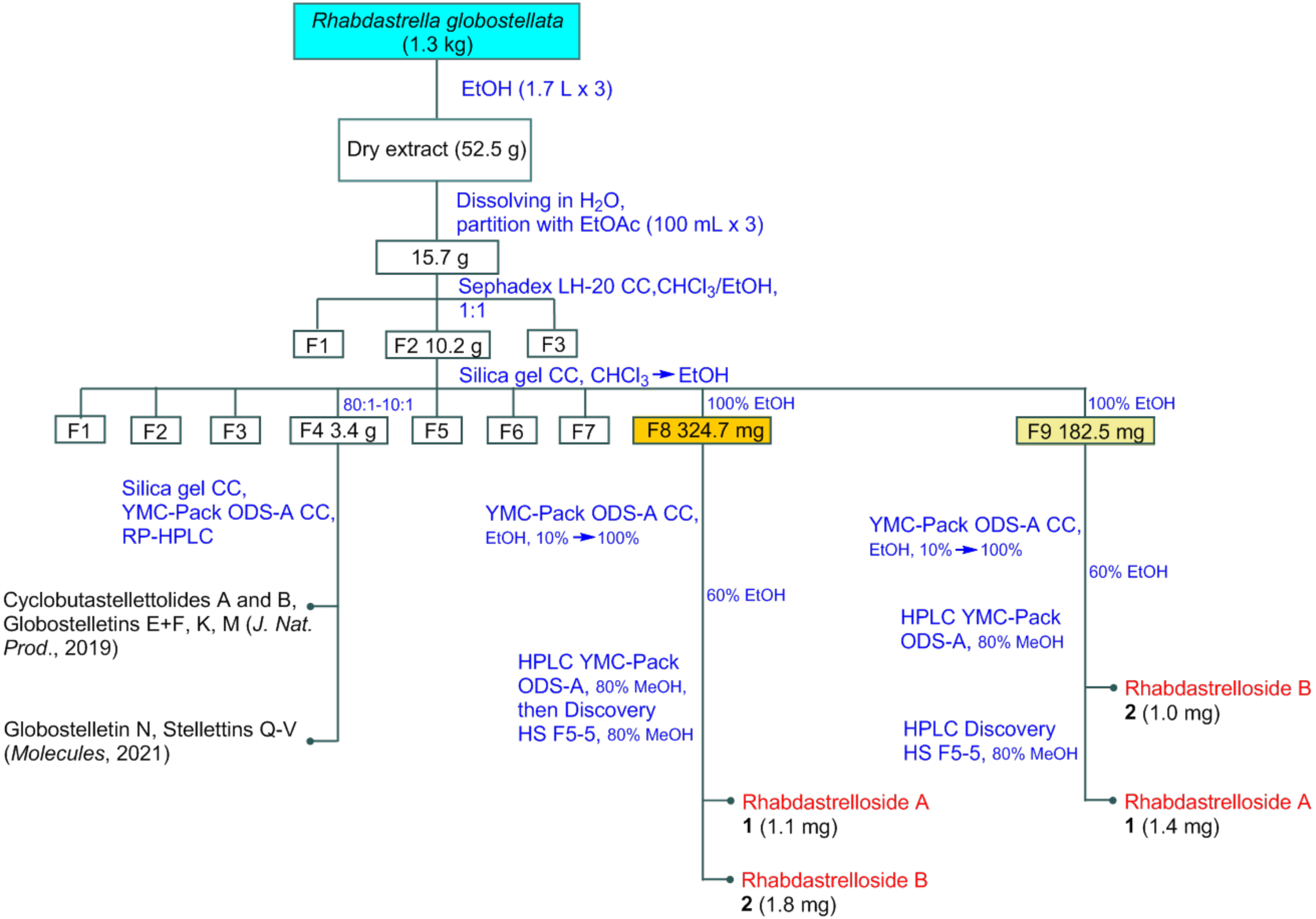

Cyclobutastellettolides A and B together with known globostelletins M, K, E, F were reported by us in **2019** (*J. Nat. Prod.*, https://doi.org/10.1021/acs.jnatprod.9b00824), and series of stellettins Q-V, along with known globostellettin N, was described in **2021** (*Molecules*, https://doi.org/10.3390/molecules26030678).
